# *LCORL* and *STC2* Variants Increase Body Size and Growth Rate in Cattle and Other Animals

**DOI:** 10.1093/gpbjnl/qzaf025

**Published:** 2025-03-17

**Authors:** Fengting Bai, Yudong Cai, Min Qiu, Chen Liang, Linqian Pan, Yayi Liu, Yanshuai Feng, Xuesha Cao, Qimeng Yang, Gang Ren, Shaohua Jiao, Siqi Gao, Meixuan Lu, Xihong Wang, Rasmus Heller, Johannes A Lenstra, Yu Jiang

**Affiliations:** Key Laboratory of Animal Genetics, Breeding and Reproduction of Shaanxi Province, College of Animal Science and Technology, Northwest A&F University, Yangling 712100, China; Key Laboratory of Animal Genetics, Breeding and Reproduction of Shaanxi Province, College of Animal Science and Technology, Northwest A&F University, Yangling 712100, China; Key Laboratory of Animal Genetics, Breeding and Reproduction of Shaanxi Province, College of Animal Science and Technology, Northwest A&F University, Yangling 712100, China; Key Laboratory of Animal Genetics, Breeding and Reproduction of Shaanxi Province, College of Animal Science and Technology, Northwest A&F University, Yangling 712100, China; College of Animal Science and Technology, Northwest A&F University, Yangling 712100, China; College of Veterinary Medicine, Northwest A&F University, Yangling 712100, China; Key Laboratory of Animal Genetics, Breeding and Reproduction of Shaanxi Province, College of Animal Science and Technology, Northwest A&F University, Yangling 712100, China; Key Laboratory of Animal Genetics, Breeding and Reproduction of Shaanxi Province, College of Animal Science and Technology, Northwest A&F University, Yangling 712100, China; Key Laboratory of Animal Genetics, Breeding and Reproduction of Shaanxi Province, College of Animal Science and Technology, Northwest A&F University, Yangling 712100, China; College of Animal Science and Technology, Northwest A&F University, Yangling 712100, China; Key Laboratory of Animal Genetics, Breeding and Reproduction of Shaanxi Province, College of Animal Science and Technology, Northwest A&F University, Yangling 712100, China; Key Laboratory of Animal Genetics, Breeding and Reproduction of Shaanxi Province, College of Animal Science and Technology, Northwest A&F University, Yangling 712100, China; Key Laboratory of Animal Genetics, Breeding and Reproduction of Shaanxi Province, College of Animal Science and Technology, Northwest A&F University, Yangling 712100, China; Key Laboratory of Animal Genetics, Breeding and Reproduction of Shaanxi Province, College of Animal Science and Technology, Northwest A&F University, Yangling 712100, China; Department of Biology, University of Copenhagen, 2200 Copenhagen, Denmark; Faculty of Veterinary Medicine, Utrecht University, 3584 CM, Utrecht, The Netherlands; Key Laboratory of Animal Genetics, Breeding and Reproduction of Shaanxi Province, College of Animal Science and Technology, Northwest A&F University, Yangling 712100, China

**Keywords:** Common improving gene, Convergent selection, *LCORL*, *STC2*, Ancestral recombination graph

## Abstract

Natural variants can significantly improve growth traits in livestock and serve as safe targets for gene editing, thus being applied in animal molecular design breeding. However, such safe and large-effect mutations are severely lacking. Using ancestral recombination graphs, we investigated recent selection signatures in beef cattle breeds, pinpointing sweep-driving variants in the *LCORL* and *STC2* loci with notable effects on body size and growth rate. The ACT-to-A frameshift mutation in *LCORL* occurs mainly in central-European cattle, and stimulates growth. Remarkably, convergent truncating mutations were also found in commercial breeds of sheep, goats, pigs, horses, dogs, rabbits, and chickens. In the *STC2* gene, we identified a missense mutation (A60P) located within the conserved region across vertebrates. We validated the two natural mutations in gene-edited mouse models, where both variants in homozygous carriers significantly increase the average weight by 11%. Our findings provide insights into a seemingly recurring gene target of body size enhancing truncating mutations across domesticated species, and offer valuable targets for gene editing-based breeding in animals.

## Graphical abstract

**Graphical abstract:**
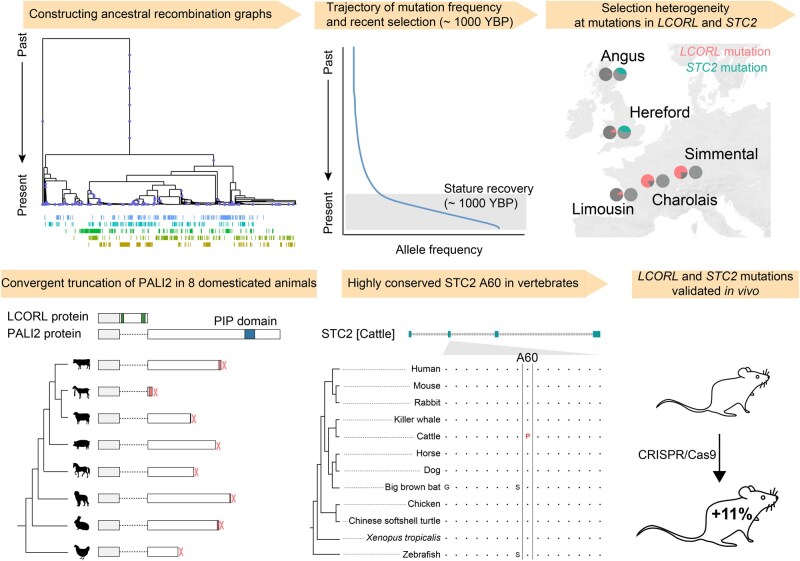

## Teaser

• The ancestral recombination graph of cattle was reconstructed and allele frequency trajectories were traced to identify mutations with selection heterogeneity between continental European and British beef cattle.
• *LCORL* and *STC2* mutations were identified as candidates with convergent selection patterns across domesticated species.
• Functional validation was performed on *LCORL* and *STC2* mutations using gene-edited mouse models to assess their role in trait variation.

## Introduction

**Figure 1:**
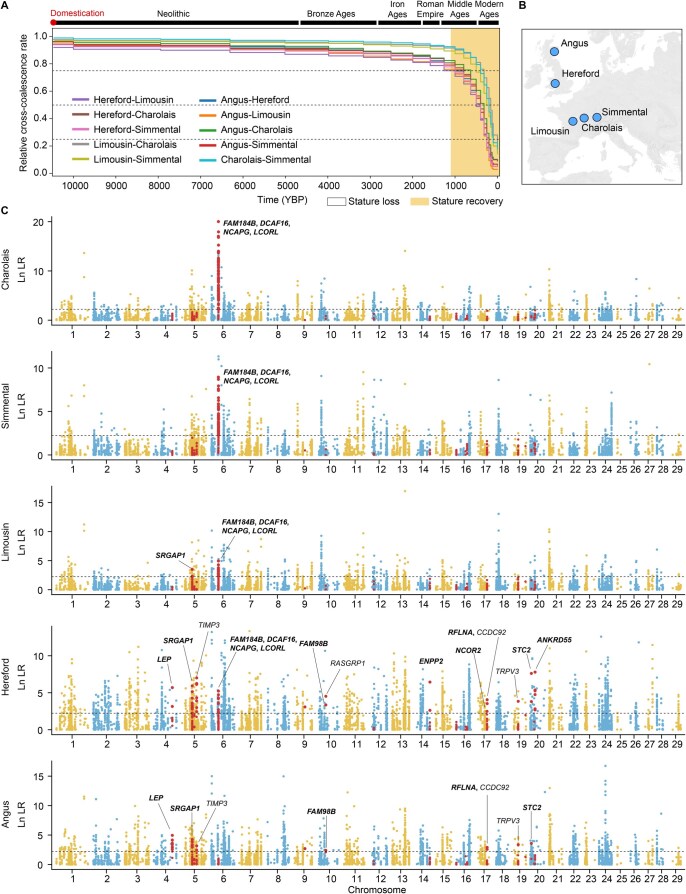
Divergence time of five European beef cattle breeds and selection analysis over the past 1000 years **A**. Relative cross-coalescence rates among the five beef cattle breeds inferred from the ARG. Around 1000 years ago, genetic divergence among the breeds accelerated, coinciding with a gradual recovery in the body size of domestic cattle. **B**. Geographic origins of the five European beef cattle breeds studied in this research. **C**. CLUES analyses of highly differentiated biallelic SNVs across the five cattle breeds. Genes overlapping or nearest of the CLUES-selected SNVs are annotated. Selected genes associated with human body size are in bold. The black dashed line represents the ln LR threshold for rejecting neutral mutations. Variants located within cattle body size QTLs are highlighted in red. ARG, ancestral recombination graph; YBP, years before present; SNV; single nucleotide variant; LR, likelihood ratio; QTL, quantitative trait locus.

Body size and growth rate are critical selection criteria in domesticated animals, particularly for meat-producing livestock. Various livestock breeds exhibit together substantial phenotypic diversity, providing an excellent resource for exploring large-effect variants contributing to differences in body size. European cattle body size has increased over the past 1000 years (**Figure 1**A) [1]. Because mammals share a conserved set of genes regulating body size [2,3], investigating large-effect variants in cattle may have broad applications for gene editing-based improvement in other domesticated species as well as for human health.

Domestic taurine cattle originated from aurochs in the Fertile Crescent around 10,500 years ago [4,5] and were introduced to Europe approximately 8500 years ago [6,7]. Archaeological evidence suggests that after domestication, the body size of cattle decreased until the Middle Ages, after which it gradually increased [1], indicating selection for larger body size, which could have left signatures of selective sweeps in the genome. Concordantly, some cattle body size and body weight quantitative trait loci (QTLs) identified through genome-wide analysis also exhibit signatures of selective sweeps [2]. Cattle body size traits thus present an ideal and valuable model for integrating genome-wide association studies (GWASs) with selective sweep analyses to identify sweep-driving mutations. However, previous selective sweep analyses of body size QTLs have generally focused on genomic regions rather than individual variants, limiting the resolution in localizing adaptive mutations [2]. So far, several candidate gene variants have been identified in Australian cattle [8], Qinchuan cattle [9], Wagyu [10], Gelbvieh [11], Canadian beef breeds [12], Red Angus [13] and Belgian Blue cattle [14]. With the exception of An et al. [10], these studies consistently localize a causative variant to bovine chromosome 6 within or near the *NCAPG* and *LCORL* genes together with several other variants. A haplotype encompassing two *LCORL* coding variants was proposed to be associated with increased lean growth in the Charolais breed [15].

In this study, we focused on five beef cattle breeds: Angus and Hereford from Britain, and Charolais, Limousin, and Simmental from Continental Europe. These breeds exhibit systematic differences in body size and weight, with British cattle generally being smaller and lighter compared to Continental European cattle [16,17]. Utilizing the advanced ancestral recombination graph (ARG) [18] combined with the CLUES analyses [19], which facilitates selection scans at the single nucleotide variant (SNV) level, we screened for highly differentiated variants between Britain and Continental Europe cattle to assess evidence of selection within the last millennium. We identified several variants likely undergoing selective sweeps and contributing to population differentiation in body size between British and Continental beef cattle. Subsequently, we focused on two gene loci that exhibited the highest significance of recent selection and a substantial impact on growth rate. By integrating *trans*-ancestral selective sweep analysis, we pinpointed the variants most likely driving selective sweeps in these regions. Two new coding variants in the *LCORL* and *STC2* genes were functionally validated in gene-edited mice. For *LCORL,* convergent selection was indicated by similar variants in seven other domestic species.

## Results

### Analysis of recent selection based on ARG

In this study, we constructed ARG using SNVs and estimated relative cross-coalescence rates among five European beef cattle breeds: Angus, Hereford, Limousin, Simmental, and Charolais (Figure 1B). The relative cross-coalescence rate displayed a decline to 0.5 between British cattle and Continental European cattle at 631 years ago (cross-coalescence rates from 0.75 at 1259 years ago to 0.25 at 200 years ago) (Figure 1A). This suggests that recent strong artificial selection has driven rapid genetic divergence among these breeds. We used *F*st statistics to assess the degree of differentiation for each biallelic SNV between British and central-continental breeds (Figure S1), defining the top 0.05% of SNVs (12,753 in total) as highly differentiated SNVs. We used CLUES to reconstruct frequency trajectories of SNVs across the five breeds and calculated the likelihood ratio (ln LR) for selection *vs.* neutrality over the last 1000 years (Figure 1C). To reduce false selection signals due to genetic drift, we simulated the demographic history of the five beef cattle and established the ln LR threshold for rejecting neutrality (cutoff = 2.23) (Figure S2). This allowed the selection of 3208 SNVs from 12,753 highly differentiated SNVs located in 354 loci (Figure 1C; Table S1).

In order to correlate these SNVs with body size traits, either as causal variants or in strong linkage disequilibrium (LD) with causal variants, we compared their locations with those of 158 known cattle body size QTLs [2] (Table S2). We found 111 CLUES-identified selective SNVs located within 11 body size QTLs (Figure 1C; Table S3). Of the 18 genes overlapping or nearest of the 111 CLUES-identified selective SNVs, 12 were found to be associated with body height in humans [20,21], more than expected by chance (*P* < 4 × 10^−9^, chi-squared test). The most significant associations were found for the body-size QTL regions containing the *NCAPG-LCORL* and *STC2* loci. Notably, in a previously reported GWAS of average daily gain in a mixed population comprising these five cattle ancestries [12], the *NCAPG-LCORL* and *STC2* loci were identified as the first and third most significant loci, respectively, with effect sizes of 71 g/day and 31 g/day. This suggests large effects on both body size and growth rate. Consequently, we conducted further in-depth analyses of the *NCAPG-LCORL* and *STC2* loci to investigate the variants driving their selective sweeps and population differentiation.

### Identification of the sweep-driving mutation at the *NCAPG-LCORL* locus

**Figure 2:**
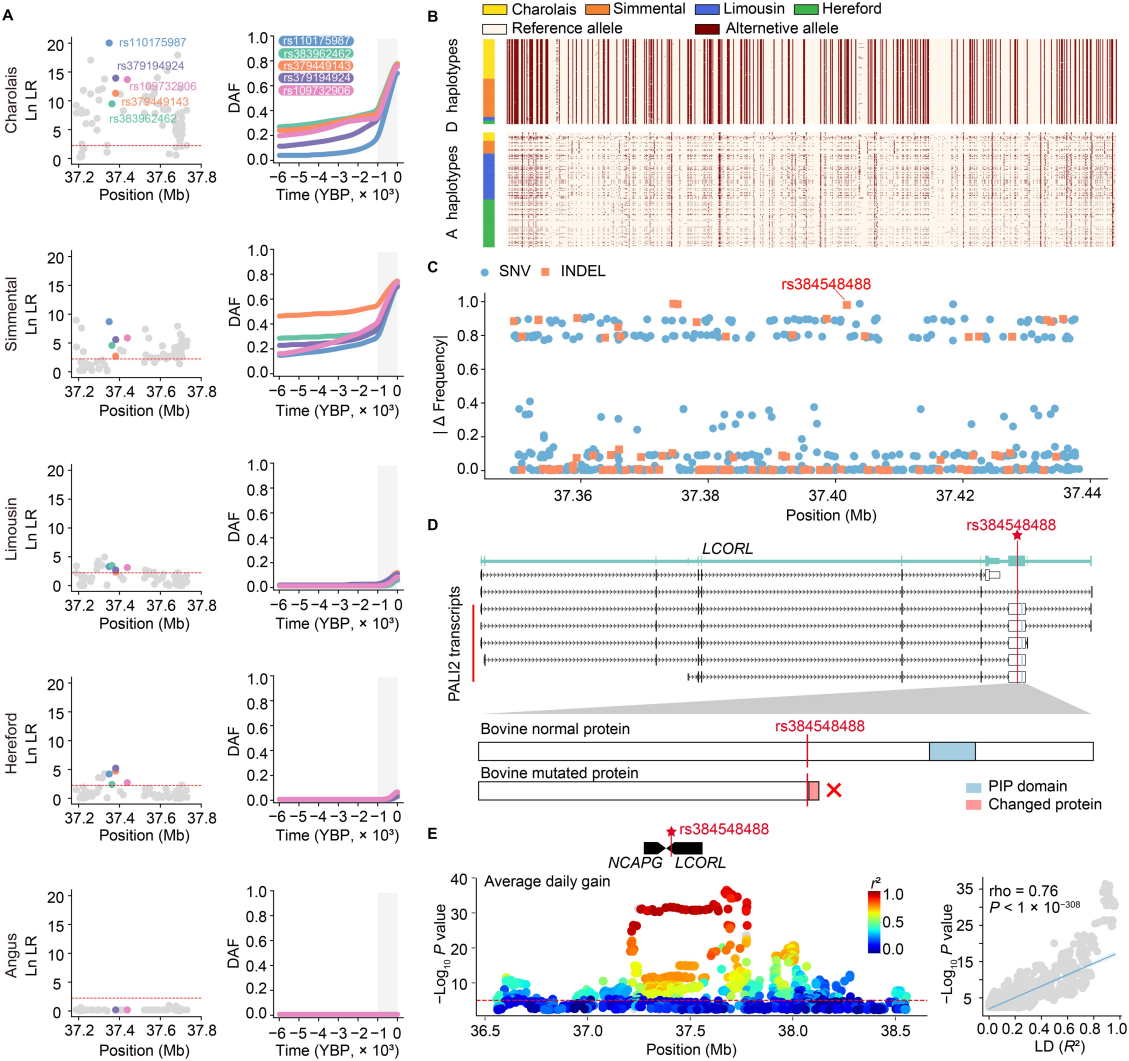
The rs384548488 mutation at *LCORL* is a candidate variant for cattle growth in the *NCAPG-LCORL* region **A**. Detailed plots for Chr6:37,180,233–37,817,575 encompassing the *NCAPG-LCORL* locus. The left column presents zoomed-in Manhattan plots of ln LR for each breed. The right column shows allele frequency trajectories for the five shared selective SNVs in the CLUES analysis. Gray shading highlights the time range from 1000 years ago to the present. **B**. A clear difference is observed between D and A haplotypes, and the notably lower diversity of D haplotypes suggests a selective sweep. The alternative allele relative to the reference genome is indicated in red. D haplotypes (*n* = 315), A haplotypes (*n* = 425). **C**. The absolute frequency difference between D and A haplotypes. SNVs are represented by circles, while INDELs are represented by squares. **D**. Schematic of the *LCORL* gene structure and its transcripts. The frameshift mutation (rs384548488) is highlighted by a red vertical line. Below the gene structure is a schematic of the truncation in the PALI2 protein, an isoform expression product of *LCORL*, caused by rs384548488. **E**. The rs384548488 variant is associated with cattle average daily gain variation. The left plot shows the *NCAPG-LCORL* locus as a QTL for average daily gain [12]. The red line is the nominal significance at *P* = 1 × 10^−5^. The right plot demonstrates a significant positive correlation between the −log_10_  *P* value of variants in average daily gain GWAS and their LD (*R*^2^) with rs384548488, indicating that rs384548488 is linked to growth traits. DAF, derived allele frequency; INDEL, insertion and deletion; GWAS, genome-wide association study; LD, linkage disequilibrium; PIP, PALI interaction with PRC2; PALI2, PRC2-associated LCORL isoform 2.

The *NCAPG-LCORL* region, located in cattle body size QTL Chr6:37,180,233–37,768,454, was identified as the most significant region in our CLUES analysis for Charolais and Simmental cattle (Figure 1C), with the selected mutations showing high frequency (frequency ≥ 0.69) (**Figure 2**A; Table S3). However, in Angus and Hereford cattle from Britain, there is no significant evidence of selection, or the putatively selected allele remains at low frequency (frequency ≤ 0.1) (Figure 2A; Table S3). Within the Chr6:37,180,233–37,768,454 region, 85 SNVs exceeded the genome-wide *F*st threshold (top 0.05%), 65 SNVs of which displayed CLUES-identified selection signals that were prioritized for further analysis. We hypothesize that the sweep-driving allele in this region has a single common origin across different breeds. The spread of this allele to various breeds has resulted in heterogeneous LD patterns with the sweep-driving allele. Consequently, the true sweep-driving allele should exhibit selection signals in all four breeds that show evidence of selective sweep in the *NCAPG-LCORL* region. In contrast, alleles that have been hitchhiked under genetic drift may only show selection signals in certain breeds. Therefore, *trans*-ancestral analyses that combine CLUES results from different breeds can aid fine-mapping by capitalizing on ancestral differences in LD patterns. Among CLUES-identified selected SNVs, five SNVs spanning 89 kb (Chr6:37,349,373–37,438,248) shared CLUES selection signals among these four breeds that show selective sweep in the *NCAPG-LCORL* region (Figure 2A; Table S3). These five shared selected mutations define a narrower selection-targeted haplotype. Across the four breeds exhibiting selective sweep signals at the *NCAPG-LCORL* locus, 315 haplotypes (D haplotypes) carry the derived alleles of these five shared mutations, while 425 haplotypes (A haplotypes) carry the ancestral alleles (Figure 2B). The D haplotypes are mainly found in Charolais and Simmental cattle, but not in Angus (Figure S3). Between the A haplotypes and D haplotypes, in addition to the five shared selected SNVs, three insertions and deletions (INDELs) also exhibited the top frequency differences (Δ Frequency = 0.98–0.99) (Figure 2C; Table S4). Among the identified variants, all five shared selected SNVs and two of the three top differential INDELs were located in non-coding regions lacking known regulatory elements (Figure S4), and the remaining one is a frameshift mutation (rs384548488, ACT-to-A) (Table S5). The 2-bp mutation on rs384548488 is located in *LCORL* and leads to the truncation of its isoform PRC2-associated LCORL isoform 2 (PALI2), resulting in the complete loss of the PALI interaction with PRC2 (PIP) domain in PALI2 (Figure 2D) [22]. Using the GWAS results for body weight in Red Angus and average daily gain in a mixed cattle population [12,13], we inferred that the level of LD (*R*^2^) with rs384548488 is strongly correlated (0.67 ≤ rho ≤ 0.76, *P* < 1 × 10^−25^) with the −log_10_-transformed GWAS *P* values of the variants (Figure 2E, Figure S5). This suggests that rs384548488 is strongly associated with growth trait variations in several beef populations (see below for its geographic range). Therefore, rs384548488*A, a predicted loss-of-PIP-domain (pLoPD) mutation, is highly likely the sweep-driving allele and the causal variant affecting cattle body size and growth rate at the *NCAPG-LCORL* locus.

### A pLoPD mutation similar to rs384548488*A increases body size and weight in mice

**Figure 3:**
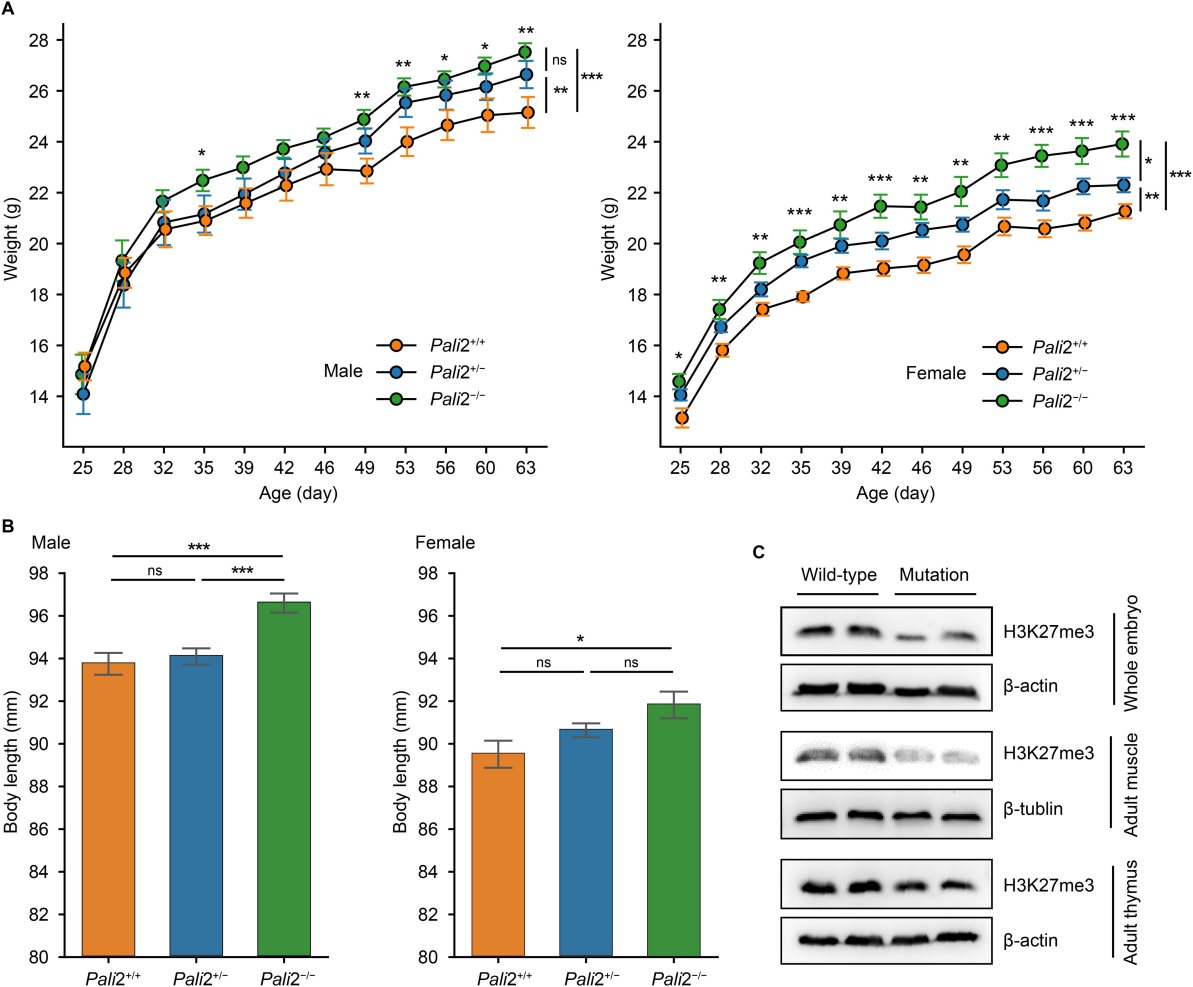
The pLoPD mutation increases body weight and body length in mice **A**. Total body weight of wild-type (+/+), heterozygous (+/−), and homozygous (−/−) PALI2 PIP domain knockout mice from 3 to 9 weeks of age. Number of male mice in each genotype: wild-type (*n* = 10), heterozygous (*n* = 10), and homozygous (*n* = 10). Number of female mice in each genotype: wild-type (*n* = 10), heterozygous (*n* = 9), and homozygous (*n* = 9). Error bars represent mean ± SE. Significant difference in body weight between *Pali2*^−/−^ and *Pali2*^+/+^ mice at each time point was determined by two-sided Student’s *t*-test (notations above each time point). Significant difference in body weight between the three genotypes of male mice (49 to 63 days) or female mice (25 to 63 days) was determined by two-way ANOVA followed by Tukey’s post hoc test (notations on the far right). **B**. Body length of *Pali2*^+/+^, *Pali2*^+/−^, and *Pali2*^−/−^ mice at 7 weeks of age. Number of male mice in each genotype: wild-type (*n* = 14), heterozygous (*n* = 23), and homozygous (*n* = 19). Number of female mice in each genotype: wild-type (*n* = 8), heterozygous (*n* = 32), and homozygous (*n* = 9). Error bars represent mean ± SE. Significant difference was determined by one-way ANOVA followed by Tukey’s post hoc test. **C**. Western blot analysis of H3K27me3 using the whole-cell lysates from wild-type and mutant mouse embryos at E14 and from muscle and thymus tissues of wild-type and mutant adult mice, probed with the indicated antibodies. *, *P* < 0.05; **, *P* < 0.005; ***, *P* < 0.0005; ns, not significant. pLoPD, predicted loss-of-PIP-domain; ANOVA, analysis of variance; SE, standard error.

To test this hypothesis, we used CRISPR-Cas9 to introduce a pLoPD mutation (Chr5:45,882,460–45,882,553del, 94-bp deletion) in C57BL/6J mice, mirroring the rs384548488 mutation in cattle (Figures S6 and S7). Through thymus tissue RNA-seq analysis, we identified the presence of the pLoPD mutation in the PALI2 transcript, while the overall expression level of *Lcorl* remained unaltered (Figure S8). We weighed wild-type (+/+), heterozygous (+/−), and homozygous (−/−) PALI2 PIP domain knockout mice from weaning to 9 weeks of age (**Figure 3**A). The *Pali2*^−/−^ mice are significantly heavier than *Pali2*^+/+^ mice. The effect of the pLoPD mutation on body weight displayed a dosage-dependent trend. At 9 weeks of age, *Pali2*^+/−^ and *Pali2*^−/−^ males were respectively 5.9% (1.5 g) and 9.4% (2.4 g) heavier than *Pali2*^+/+^ males. Similarly, *Pali2*^+/−^ and *Pali2*^−/−^ females were respectively 4.8% (1.0 g) and 12.4% (2.6 g) heavier than *Pali2*^+/+^ females. At 7 weeks of age, the body length of *Pali2*^−/−^ males and females was also significantly increased, with a 3.0% (2.9 mm) and 2.6% (2.3 mm) increase, respectively, compared to wild-type mice (Figure 3B). We next analyzed mouse embryos at embryonic day 14 (E14), as well as on muscle and thymus tissues from adult mice by Western blot, to determine whether increased growth rates are related to changes in H3K27me3 levels (Figure 3C). The H3K27me3 levels were found to be reduced in *Pali2*^−/−^ mice compared to wild-type mice. This result confirms the possible function of PALI2 as a crucial component of the PRC2 complex [22], which mediates H3K27me3 in regulating individual development and growth [23–25].

### Convergent artificial selection of pLoPD mutation in eight domesticated animals

**Figure 4:**
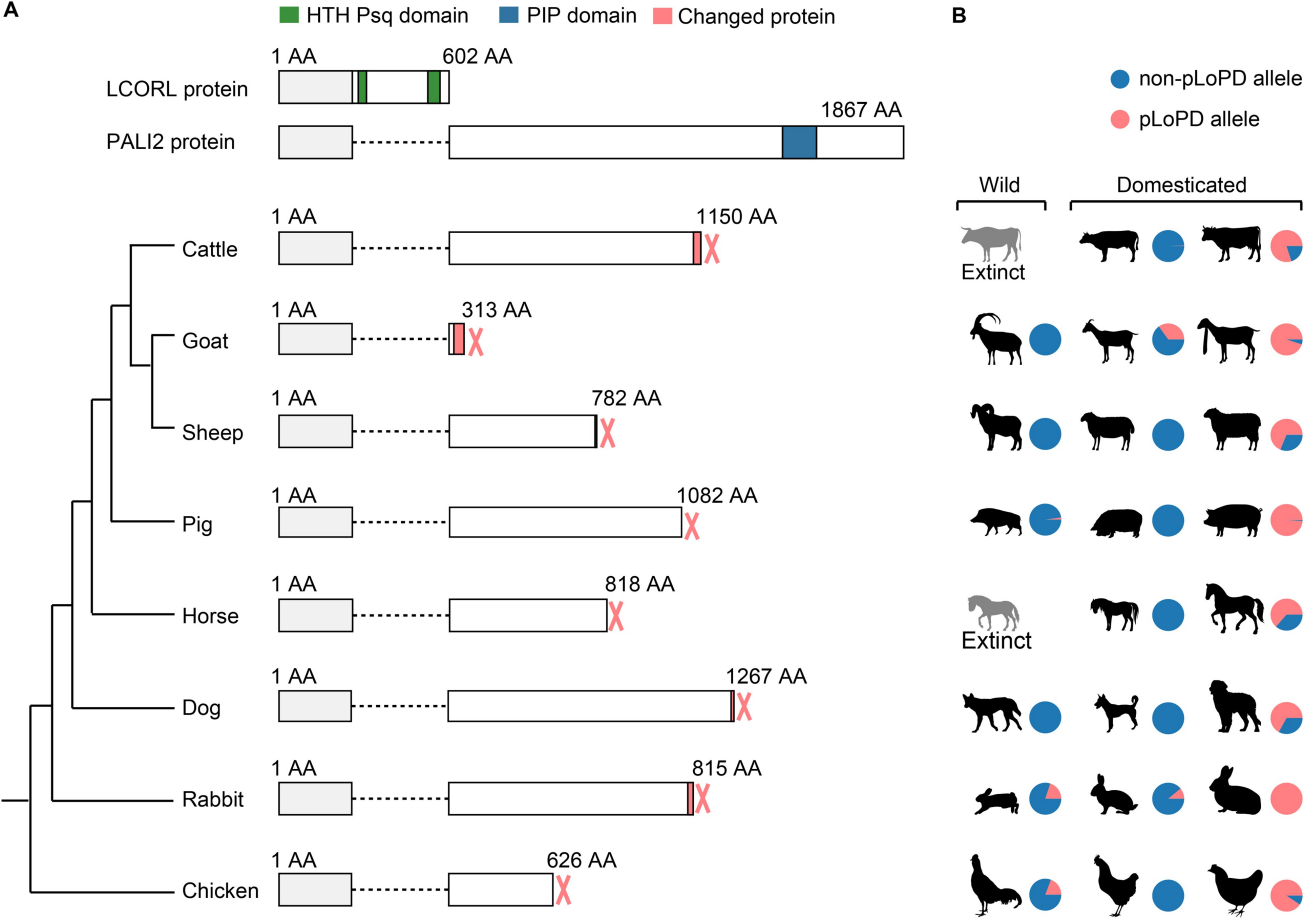
The loss of the PIP domain in PALI2 across eight domesticated animals **A**. Schematic representations of the LCORL and PALI2 proteins, two products encoded by the *LCORL* gene. The phylogenetic tree is based on the UCSC 100 Vertebrates dataset. Natural pLoPD mutations are observed in cattle, goats, sheep, pigs, horses, dogs, rabbits, and chickens, without affecting the LCORL protein. The HTH Psq domain, unique to the LCORL protein, is highlighted in green, while the PIP domain, specific to the PALI2 protein, is shown in blue. The regions altered by pLoPD mutations are marked in red, and the shared regions between the two forms are colored in gray. **B**. Distribution of pLoPD mutations in wild species and domesticated breeds or populations. The wild ancestors include the extinct wild ancestors for cattle and horses, the bezoar for goats, the Asian mouflon for sheep, the Eurasian wild boar for pigs, the gray wolf for dogs, the European wild rabbit for rabbits, and *Gallus gallus spadiceus* for wild chicken. The domesticated breeds or populations depicted are: Cattle — the Angus cattle, a European small beef breed, and the Charolais cattle, a European large beef breed; Goat — two types of Pakistani goat populations reported by Saif et al. [30]: the Pakistani goat breeds with and without the selection signature at the *LCORL* locus; Sheep — the Hu sheep, an Asian local breed, and the Merino sheep, a European commercial breed; Pig — the Meishan pig, an Asian local breed, and the Duroc pig, a European commercial meat breed; Dog — small breeds (weight < 10 kg) and large breeds (weight > 41 kg) based on the report by Plassais and colleagues [3]; Rabbit — the Dutch and Flemish Giant rabbits; Chicken — the commercial egg-laying White Leghorn and the commercial meat-producing White Plymouth Rock chickens. pLoPD refers to alleles predicted to result in the loss of the PIP domain in PAIL2, while non-pLoPD refers to alleles without such predicted effects. Detailed population and breed information is provided in Tables S12 and S13. AA, amino acid.

Interestingly, we observed that several other domestic animals exhibit the loss of the PIP domain in PALI2 (**Figure 4**). Reference genomes of domestic pigs (Sscrofa11.1), sheep (ARS-UI_Ramb_v2.0), and rabbits (OryCun2.0) all have a pLoPD mutation upstream of the PIP domain in PALI2 (Figures S9 and S10). Comparing domestic and wild populations suggests a selective sweep in the *LCORL* locus in European domestic pigs and rabbits [26,27]. However, these studies have not pinpointed the sweep-driving variants within the *LCORL* locus.

In pigs, the pLoPD mutation is Chr8:g.12,829,718T [6] (ancestral allele: Chr8:g.12,829,718T [7], rs697231176). The pLoPD mutation also exhibited differentiation between wild boars and European domestic pigs (Figure S11; Table S6). The pLoPD allele at rs697231176 is found only in one Ukrainian wild boar but is absent in all 43 other European and Asian wild boars (Figure S11A). In domestic pigs, the pLoPD allele at rs697231176 is nearly fixed in major European meat breeds (Figure S11B).

In rabbits, the pLoPD mutation is Chr2:g.8,433,339 (ancestral allele: Chr2:g.8,433,339del). The frequency of the pLoPD allele is higher in domestic European rabbits compared to wild European rabbits, with the exception of the domestic Dutch rabbit (Figure S12; Table S7). In the Dutch rabbit, the frequency of the pLoPD allele at Chr2:g.8,433,339 is only 0.11, which correlates with the smaller body size observed in this breed.

In sheep, the pLoPD mutation is Chr6:g.38,076,935_38,076,936 (ancestral allele: Chr6:g.38,076,935_38,076,936insCCTGGTGGTA). The pLoPD allele is absent in wild sheep (Figure S13A; Table S8). Moreover, extended haplotypes carrying the pLoPD allele are longer than those with the ancestral allele in Merino sheep (Figure S13B).

We observed the same occurrences in domesticated chickens (Figure S14). Reference genomes GRCg7b and GRCg6a lack annotations for LCORL isoforms containing the PIP domain. However, PALI2 has been successfully annotated in the reference genome GRCg7w (Figure S14). We hypothesize that pLoPD variants in reference genomes has led to incorrect annotations. According to the pangenome representing the genomes of 30 chickens [28], a nonsense variant rs317817652 (NC_052576.1:g.75386062C>T, XP_046772233.1:p.Arg626Ter) results in the complete loss of the PIP domain in chicken PALI2 (Figure S14). Confirming our hypothesis, reference genomes GRCg7b and GRCg6a both carry the rs317817652*T nonsense mutation. The pLoPD mutation rs317817652*T is also common in the wild species *Gallus gallus spadiceus* (frequency = 0.19), from which domesticated chickens are derived (Table S9). However, this variant is absent in the other four subspecies of red jungle fowl. A GWAS on body weight and size in Chinese local chickens [29] shows that rs317817652*T significantly increases body weight and size (Table S10). However, because the analysis was based on the reference genome GRCg6a, rs317817652 was annotated only as a non-coding variant.

We also found pLoPD mutations in goats, horses, and dogs (Figure 4; Table S11). Previous studies have reported that pLoPD mutations are associated with increased body size in these species [3,30,31]. The loss of the PIP domain in PALI2 in domestic cattle, goats, sheep, horses, pigs, dogs, rabbits, and chickens suggests convergent artificial selection of large body size and fast growth rates during animal domestication.

### Two candidate variants underlie genetic associations in the *STC2* locus

**Figure 5:**
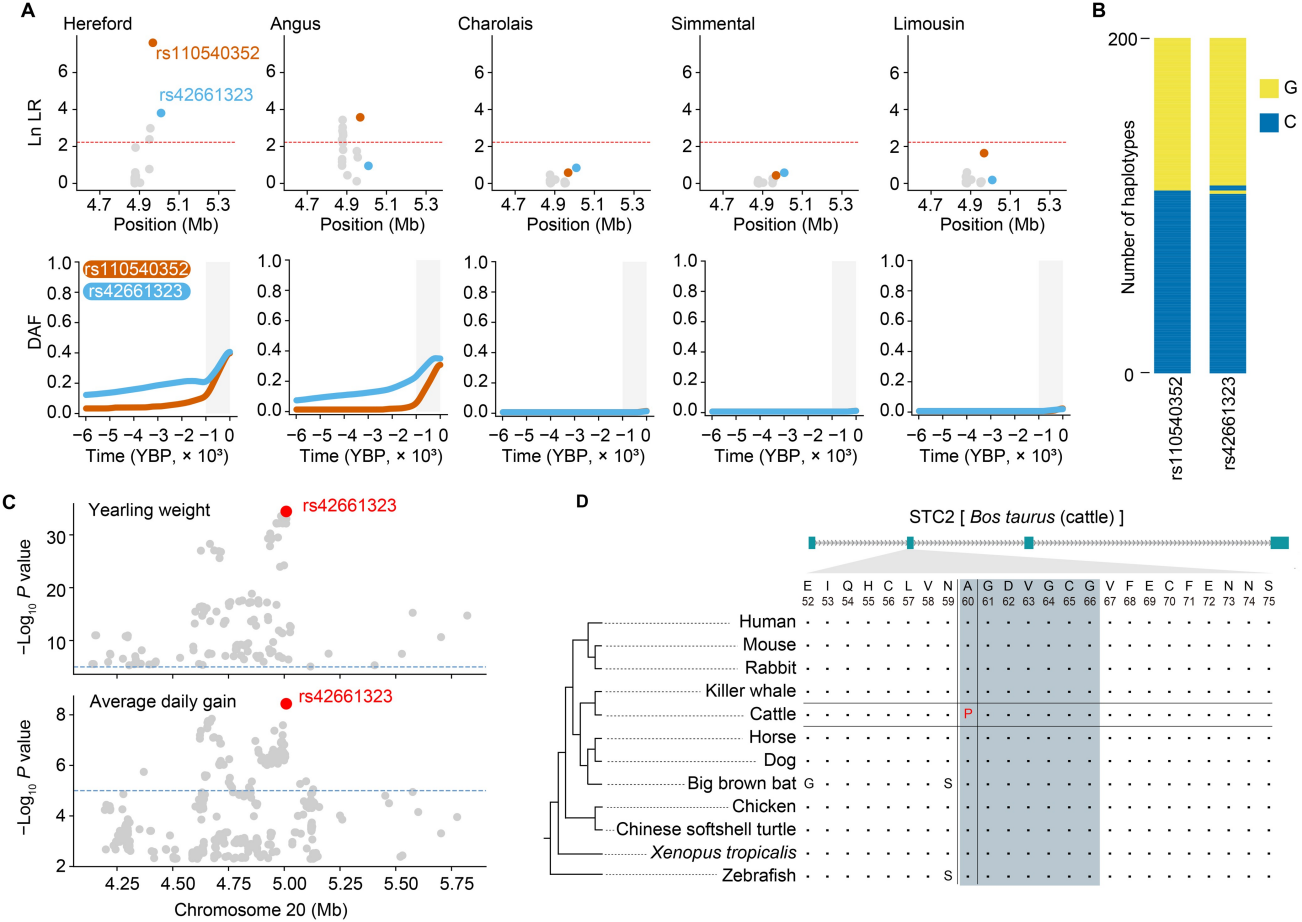
Selective sweep and functional analysis of ***STC2*** variants **A**. Detailed plots for Chr20:4,598,655–5,366,902, containing the *STC2* locus. The upper row shows zoomed-in Manhattan plots of ln LR for each breed. The lower row shows allele frequency trajectories for the top SNVs in the CLUES analysis and the missense SNVs. Gray shading indicates the time range from 1000 years ago to the present. **B**. Stacked bar plot of the haplotypes comprising rs110540352 and rs42661323 in the Hereford cattle (*n* = 200 haplotypes). The two derived alleles (rs110540352*G and rs42661323*G) are almost always inherited together as a single haplotype. The y-axis represents the number of haplotypes, and the x-axis represents the indicated SNVs. **C**. rs42661323 is the lead GWAS SNV for yearling weight and average daily gain in cattle. The GWAS data source is the same as in Figure 2C and Figure S5. **D**. Conservation analysis of the amino acid residue at position 60 of STC2. The amino acid residues at positions 60–66 of STC2 interact with PAPP-A, marked with blue shading.

*STC2* is located within the body size QTL Chr20:4,967,596–5,008,911. Within this region, only two variants, rs110540352 and rs42661323, display CLUES selection signals in Hereford and Angus (**Figure 5**A) and have the highest *F*st values (Figure S15A). In Hereford cattle, both mutations are in high LD (*R*^2^ = 0.92): 96.7% of Hereford haplotypes with the derived allele rs110540352*G also carry the derived allele rs42661323*G (Figure 5B). Notably, rs110540352 is an intergenic variant located within a regulatory element (Figure S16A), while rs42661323 is a missense mutation within the *STC2* gene. To check for potential selective INDELs that might have been overlooked, we used rs110540352 as a tag SNV to divide the 400 haplotypes from both breeds into 162 carrying rs110540352*G (derived allele) and 238 carrying rs110540352*A (ancestral allele). The results showed that the INDEL with the highest divergence between the two haplotype groups was located in an intergenic region and not within a regulatory element (Figure S17). Therefore, we prioritized rs110540352 and rs42661323 as the main variants driving the selection sweep in the *STC2* locus.

In a GWAS for cattle body size, rs110540352, 43,529 bp upstream of the *STC2* transcription start site (Figure S16A), is the lead SNV within the body size QTL Chr20:4,967,596–5,008,911, and the derived allele rs110540352*G shows a positive effect (β = +0.16 cm) on body size [2] (Table S2). We found that rs110540352 is the CLUES lead SNV in Hereford and Angus cattle, which was also indicated by CLUES selection tests on all SNVs (Figure S15B). In mice and humans, *STC2* reduces bioactivity of insulin-like growth factors (IGFs) and inhibits growth by binding to and inhibiting PAPP-A, which cleaves IGFBPs [32,33]. Therefore, we hypothesize that the C-to-G mutation at rs110540352 decreases *STC2* expression. According to functional annotations of the cattle genome in FAANG, rs110540352 is located in an active regulatory element (Chr20:4,967,400–4,969,800) in the cerebral cortex and hypothalamus [34] (Figure S16A). Additionally, the C-to-G mutation at rs110540352 disrupts the binding motif of E2F4 (Figure S16B). Dual-luciferase reporter assays show that the C-to-G mutation at rs110540352 reduces luciferase expression (Figure S16C). For Hereford cattle heterozygous for the rs42661323 missense variant, RNA-seq of the cerebral cortex shows that the derived allele rs42661323*G (*n* = 7) has a significantly lower expression level than the ancestral C allele (*n* = 16) (Binomial Test, *P* = 0.047) (Figure S16D). The high LD between rs42661323*G and rs110540352*G in Hereford implies that rs110540352 has allele-specific expression, which supports that its G allele reduces *STC2* expression.

The missense C-to-G mutation at rs42661323 causes an amino acid change from alanine (A) to proline (P) at position 60 in STC2. In a GWAS for cattle yearling weight and average daily gain, rs42661323 is the lead SNV within the body size QTL Chr20:4,967,596–5,008,911 [12,13] (Figure 5C). The mutation rs42661323*G increases yearling weight (β = +18.10 kg) and average daily gain (β = +31.34 g). STC2 A60 is highly conserved in vertebrates except coelacanth and yellowbelly pufferfish (Figure 5D, Figure S18).

### STC2 A60P increases body size and weight in mice

**Figure 6:**
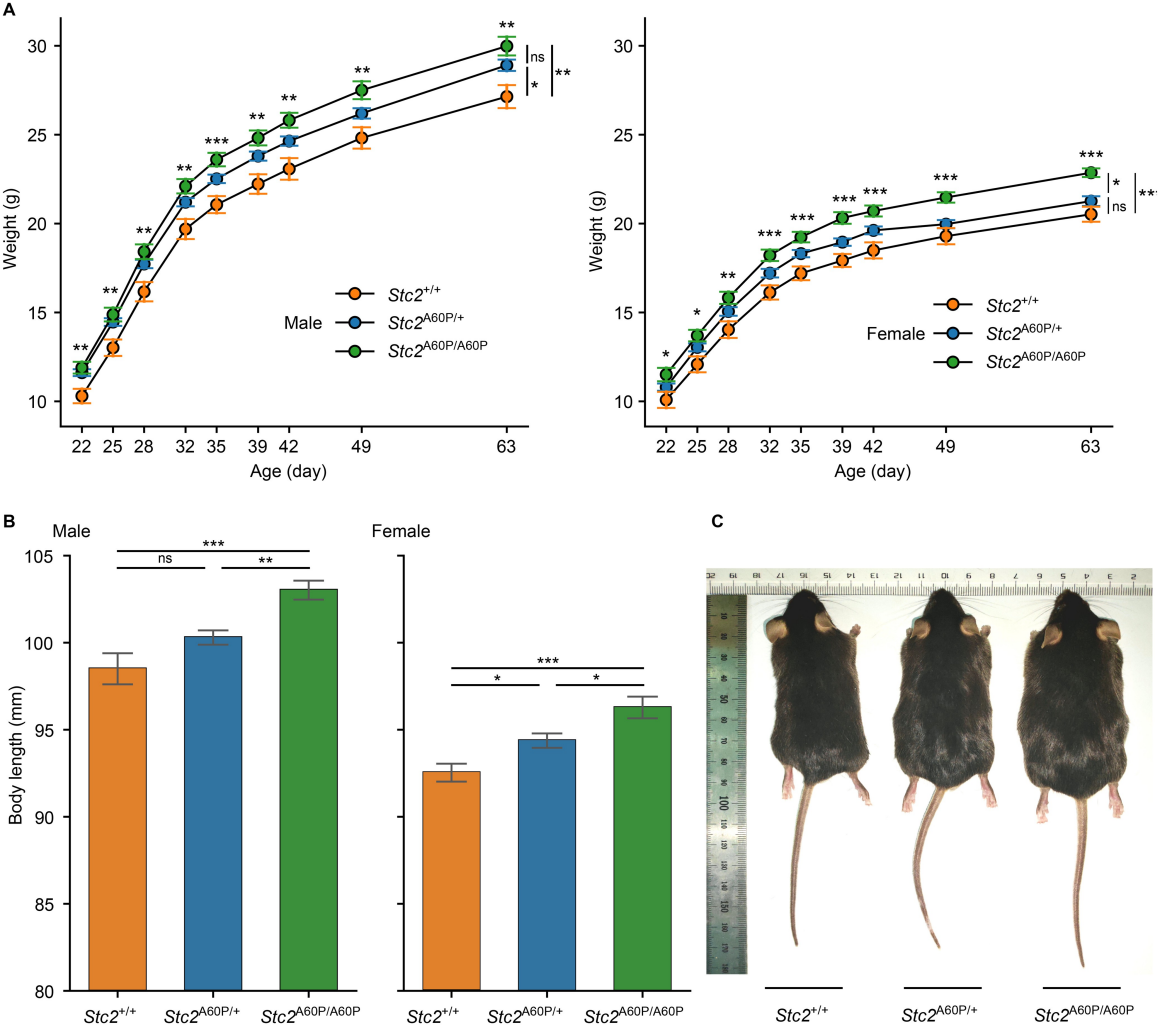
STC2 A60P increases body weight and body length in mice **A**. Total body weight of indicated littermates from 3 to 9 weeks of age. Number of male mice in each genotype: *Stc2*^+/+^ (*n* = 12), *Stc2*^A60P/+^ (*n* = 40), and *Stc2*^A60P/A60P^ (*n* = 17). Number of female mice in each genotype: *Stc2*^+/+^ (*n* = 12), *Stc2*^A60P/+^ (*n* = 30), and *Stc2*^A60P/A60P^ (*n* = 13). Error bars represent mean ± SE. Significant difference in body weight between *Stc2*^A60P/A60P^ and *Stc2*^+/+^ mice at each time point was determined by two-sided Student’s *t*-test (notations above each time point). Significant difference in body weight between the three genotypes of male mice (22 to 63 days) or female mice (22 to 63 days) was determined by two-way ANOVA followed by Tukey’s post hoc test (notations on the far right). **B**. Body length of *Stc2*^+/+^, *Stc2*^A60P/+^, *Stc2*^A60P/A60P^ mice at 14 weeks of age. Number of male mice in each genotype: *Stc2*^+/+^ (*n* = 12), *Stc2*^A60P/+^ (*n* = 40), and *Stc2*^A60P/A60P^ (*n* = 17). Number of female mice in each genotype: *Stc2*^+/+^ (*n* = 12), *Stc2*^A60P/+^ (*n* = 30), and *Stc2*^A60P/A60P^ (*n* = 13). Error bars represent mean ± SE. Significant difference was determined by one-way ANOVA followed by Tukey’s post hoc test. **C**. Representative images of littermates of *Stc2*^+/+^, *Stc2*^A60P/+^, and *Stc2*^A60P/A60P^ mice. *, *P* < 0.05; **, *P* < 0.005; ***, *P* < 0.0005; ns, not significant.

To validate the effect of the STC2 A60P mutation on body size *in vivo*, we introduced this point mutation into C57BL/6J mice using the CRISPR-Cas9 system (Figure S19). We monitored the body weight of wild-type (*Stc2*^+/+^), heterozygous (*Stc2*^A60P/+^), and homozygous (*Stc2*^A60P/A60P^) mice from weaning until 9 weeks of age (**Figure 6**A). *Stc2*^A60P/A60P^ mice are significantly heavier than *Stc2*^+/+^ mice starting at 3 weeks of age (male: *P* = 0.002; female: *P* = 0.0001; two-way ANOVA followed by Tukey’s post hoc test). The impact of the STC2 A60P mutation on body weight showed a dose-dependent trend. At 9 weeks of age, *Stc2*^A60P/+^ males and females were respectively 6.5% (1.8 g) and 3.6% (0.7 g) heavier than *Stc2*^+/+^ mice, while *Stc2*^A60P/A60P^ males and females were respectively 10.5% (2.8 g) and 11.4% (2.3 g) heavier than *Stc2*^+/+^ mice.

Consistent with our expectation, the STC2 A60P mutation also demonstrated an additive effect on body size increase (Figure 6B). At 14 weeks of age, *Stc2*^A60P/+^ males and females were respectively 1.8% (1.8 mm) and 2.0% (1.8 mm) longer than *Stc2*^+/+^ mice, whereas *Stc2*^A60P/A60P^ males and females were respectively 4.6% (4.5 mm) and 4.0% (3.7 mm) longer than *Stc2*^+/+^ mice.

### Distribution and evolution of rs384548488*A, rs110540352*G, and rs42661323*G in cattle

We investigated the origin and distribution of rs384548488*A, rs110540352*G, and rs42661323*G mutations in ancient and modern cattle. The frequencies of these three mutations in different breeds were calculated by using Run 9 of the 1000 Bull Genomes (Table S14). Most of the 478 *Bos indicus* individuals lack these three mutations except for Brahman cattle, a cross of *Bos indicus* and *Bos taurus*. Among 225 Brahman cattle, only one rs384548488*A, two rs110540352*G, and three rs42661323*G alleles were found. This suggests that all three mutations emerged after the divergence of *Bos taurus* and *Bos indicus*.

**Figure 7:**
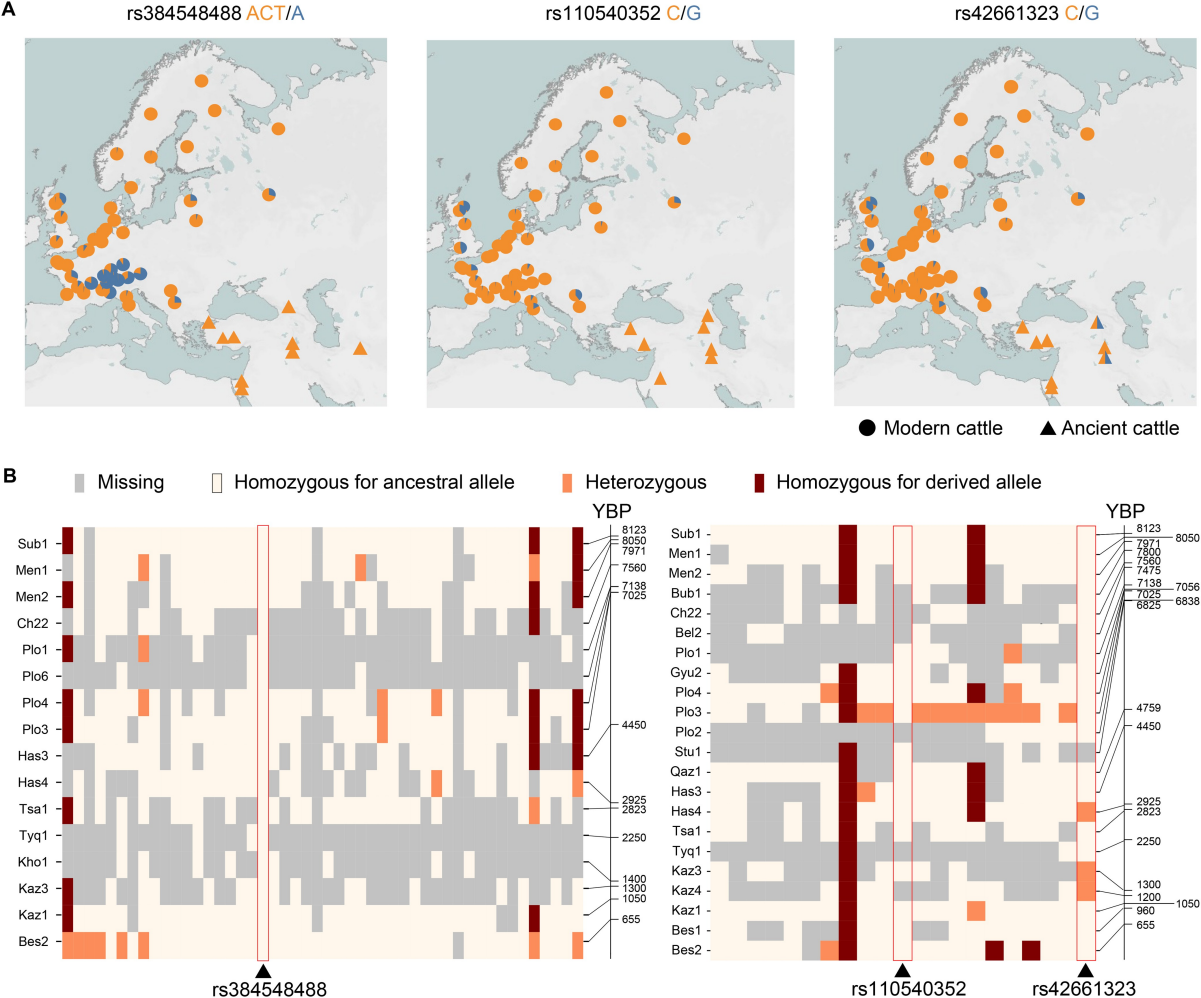
Distribution and evolution of *LCORL* and *STC2* variants in cattle **A**. Geographical distribution of erived alleles at the *LCORL* (rs384548488*A) and *STC2* (rs110540352*G and rs42661323*G) loci. Blue and orange indicate the derived and ancestral alleles, respectively. Half-orange and half-blue triangles and circles indicate the heterozygous ancient and modern cattle, respectively. The modern cattle data are from Run 9 of the 1000 Bull Genomes Project. **B**. Genotypes of *LCORL* and *STC2* loci in ancient cattle. Triangles indicate the casual variants. Missing genotypes are colored in gray, homozygotes for ancestral allele are colored in light yellow, heterozygotes are colored in orange, and homozygotes for derived allele are colored in dark red.

At the *LCORL* locus, breeds with a high frequency of the rs384548488*A allele (frequency > 0.5) predominantly originated in the Alpine region (**Figure 7**A). The rs384548488*A allele was nearly fixed in Original Braunvieh (frequency = 0.992), its related Brown Swiss (frequency = 0.997), and several other central European breeds. Remarkably, this allele was almost absent in black Angus (frequency = 0.014) but had a moderate frequency in red Angus (frequency = 0.386). However, the rs384548488*A allele was not detected in 16 ancient Middle Eastern cattle spanning 655–8123 years ago (Figure 7B). These findings suggest that rs384548488*A likely emerged after the spread of domestic *Bos taurus* from the Fertile Crescent to Europe, possibly originating in cattle of Braunvieh ancestry in the Alpine region.

At the *STC2* locus, the frequencies of both causal variants, rs110540352*G and rs42661323*G, were below 0.5 in all breeds examined (Figure 7A). Hereford cattle had the highest frequencies for both rs110540352*G and rs42661323*G. In Alpine cattle breeds where the frequency of rs384548488*A exceeded 0.5, the maximum frequencies of rs110540352*G and rs42661323*G were only 0.077, whereas these derived alleles were absent in Original Braunvieh and Brown Swiss. According to ARG, the inferred age of rs110540352*G in five beef cattle populations ranged from 911 to 1148 years ago, which was later than the inferred age of rs42661323*G ranging from 6571 to 9761 years ago. Consistent with this, rs110540352*G was absent in ancient cattle samples, while rs42661323*G was identified as heterozygous in one Iron Age and two Middle Ages ancient cattle samples (Figure 7B). Although rs110540352*G emerged later than rs42661323*G, its frequency in modern cattle was slightly higher than that of rs42661323*G. Selection coefficients inferred by CLUES analysis in Hereford cattle also indicate that rs110540352*G (s = 0.012) has undergone stronger positive selection than rs42661323*G (s = 0.007) in the last 1000 years.

## Discussion

Previous GWASs have identified a set of genes as shared regulators of body size in domesticated animals [2,3]. However, the high LD patterns posed challenges for pinpointing causal variants. The ARG encompasses the complete history of genomic mutations, facilitating detailed and robust inferences about selection [35,36]. The CLUES method, based on the ARG, has shown excellent power in inferring recent selection and fine-mapping selected sites [19]. This method is capable of estimating the onset time of a selective sweep for either selected or neutral alleles. The body size of domestic cattle exhibited a declining trend from the time of domestication until approximately 1000 years ago, after which it began to increase, particularly under the influence of artificial selection. Therefore, we propose that employing the CLUES method to constrain the selection detection interval to within the last 1000 years is an effective model for identifying the genetic variations responsible for the recent increase in the size of domestic cattle. In this study, we utilized these methods to identify 11 loci driving recent selective sweeps in body size QTLs between British and Continental beef cattle. Specifically, we proposed sweep-driving mutations at the top effect loci, *LCORL* and *STC2*. Then, we validated causal coding mutations on *LCORL* and *STC2* in gene-editing mice, which support the role of these mutations in growth. This marks the first time that causal variants regulating body size and growth traits in cattle have been experimentally verified *in vivo*. We propose that the utilization of ARG combined with the CLUES method is an effective tool for fine-mapping recently selected sites in economic QTLs of domesticated animals.

The *LCORL* locus is reported as being associated with body size in various domesticated animals, such as dogs [3], horses [31], goats [30], and cattle [2,8–15,37]. In this study, we propose that pLoPD mutations are putative sweep-driving alleles in *LCORL* in sheep, pigs, rabbits, and chickens. These results highlight its role as a convergent sweep-driving mutation across abovementioned species. Mouse models confirmed the growth-promoting effects of the pLoPD mutation in PALI2. It is important to note that PALI2 is only one type of *LCORL* expression products, and complete *Lcorl* knockout in mice leads to different outcomes [38]. The possible function of PALI2 is to enhance PRC2-mediated methyltransferase activity [22]. In mouse embryonic stem cells, PRC2 targets a range of well-known developmentally regulated genes, including the *Wnt6-Ihh* and *Hoxb* loci [25,39]. We found that the loss of the PIP domain in PALI2 has a more pronounced effect on H3K27me3 levels during embryonic development than in adult mice. This underscores the significance of PRC2-mediated methyltransferase activity in growth regulation, suggesting a particularly strong role during embryonic development.

The IGF system plays a central role in regulating overall body growth by controlling cellular growth, differentiation, and survival [40]. Consequently, numerous genes within the IGF pathway, including *IGFs*, *IGFBPs*, *GHR*, and *STC2*, are crucial for growth regulation in both humans and domesticated animals [2,3,21,41,42].

A study on human height has shown that two rare variants in human *STC2*, p.R44L and p.M86I, disrupt the inhibition of PAPP-A-mediated proteolysis of IGFBP-4 *in vitro*, significantly increasing height by 1.9 cm and 0.9 cm, respectively [33]. In cattle, we identified a causal missense mutation, rs42661323*G (STC2 p.A60P). Structural analysis of the human PAPP-A·STC2 complex [35] indicates that amino acids 60–66 in STC2 interact with PAPP-A, and STC2 A60P is located within this interaction region. Therefore, we speculate that STC2 A60P reduces STC2 binding to PAPP-A, thereby increasing IGF bioactivity and promoting growth. Consequently, saturation mutagenesis targeting the STC2-PAPP-A interaction domain via gene editing emerges as a promising strategy for generating novel and potentially advantageous *STC2* alleles. Additionally, we identified a selective non-coding mutation, rs110540352*G, downstream of the *STC2* gene, which is located in active regulatory elements in the cerebral cortex and hypothalamus. Due to the high LD between rs110540352*G and rs42661323*G, haplotypes carrying both variants are expected to have a combined effect of reducing *STC2* expression and producing functionally impaired STC2 protein.

Although this study predicts the functional mechanisms of *LCORL* and *STC2* mutations through integrated multi-omics analyses, these predictions require further experimental validation. For example, the impact of the *LCORL* frameshift mutation on PRC2-mediated H3K27me3 modification and the regulatory effect of the STC2 A60P mutation on PAPP-A binding affinity need to be further confirmed through experiments such as Co-IP and IGF bioactivity assays. Future research will focus on functional validation of these mechanisms to comprehensively reveal the biological significance of these mutations in trait regulation. Furthermore, while the gene-edited mouse models have provided valuable insights into the functional roles of these variants, we acknowledge the limitations of this approach. Species-specific differences and genetic background may influence the generalizability of our findings. To fully understand the biological significance of these variants in cattle growth and development, future studies in more relevant models or cattle populations are necessary.

The causal mutations in *LCORL* and *STC2* identified in this study are naturally occurring. The *LCORL* pLoPD mutation is a convergent sweep-driving allele observed across multiple domesticated species and even in wild populations (*e.g.*, wild rabbits and chickens). This suggests a shared genetic mechanism underlying body size variation, likely driven by artificial selection during domestication. By targeting this mutation, breeders can enhance growth traits across diverse livestock breeds. The STC2 A60P mutation, located within the interaction domain of STC2 and its ligand PAPP-A, provides a promising target for mutagenesis. This approach could generate novel advantageous alleles to optimize the IGF pathway, which plays a central role in regulating body growth, cellular differentiation, and survival. In summary, the mutations identified in this study that regulate body size and weight traits in cattle provide precise targets for genome selection and gene editing, potentially facilitating the enhancement of economically important traits in all kinds of livestock species.

## Materials and methods

### Variant processing from Run 9 of the 1000 Bull Genomes Project for ARG construction

We utilized BCFtools (v1.16) [43] for variant filtering. We excluded variants falling within the 99.90 to 100.00 tranche of the GATK Variant Quality Score Recalibration (VQSR) for single nucleotide polymorphisms (SNPs). Only biallelic SNPs with a missing rate of less than 20% were retained. To infer the ARG, we first removed singleton variants and then applied Beagle (v5.4) [44] (with parameters: window = 5, overlap = 2) for phasing the remaining variants and imputing missing variants.

### Individual selection for ARG construction

Based on the sample information provided by Run 9 of the 1000 Bull Genomes Project, the sample sizes for Angus, Hereford, Charolais, Simmental, and Limousin were 401, 142, 154, 137, and 101, respectively. To construct the ARG, we aimed to select 100 individuals from each breed.

We used KING (v2.3.0) [45] to calculate kinship coefficients between individuals (–kinship) and maximally removed closely related individuals. Detailed filtering parameters and procedures are described in File S1 Section 1. In addition to these 500 individuals from the five main breeds, we selected 168 individuals from other breeds to jointly infer the preliminary ARG.

### Determination of bovine ancestral alleles

We retained 2144 individuals with BioProject IDs from Run 9 of the 1000 Bull Genomes Project. To obtain a representative sample, we selected one individual with the highest sequencing depth from each breed, resulting in a total of 79 representative individuals.

The assignment of bovine ancestral alleles was based on a model comparison of alleles from cattle with alleles from outgroup species: Water Buffalo, Sheep, and White-Tailed Deer. While species of the *Bos* genus are more closely related and potentially more informative, they share a high proportion of genetic introgression with cattle [46].

We utilized multiple sequence alignments of 110 species (78 ruminants and 32 mammalian outgroup species), available from http://animal.omics.pro/code/index.php/RGD/loadByGet?address[]=RGD/Download/comSynDownload.php [47], to determine the alleles in Water Buffalo, Sheep, and White-Tailed Deer at each locus. We employed MafFilter (v1.3.1) [48] to generate sequence alignment files between cattle and each of the three outgroup species. We then used our custom script to retrieve sequence data for cattle and the three outgroup species in est-sfs input format. Only sites with sequence data available in at least one outgroup species were retained, resulting in 72,831,650 sites across the four species used for ancestral allele determination.

We employed the est-sfs software [49] with the K2 model to infer the probability (Pancs) of the major allele in cattle being ancestral. Alleles were determined to be ancestral if they were the major allele at a site with Pancs > 0.8 or the minor allele at a site with Pancs < 0.2. For the remaining sites where ancestral alleles could not be determined, we used the reference allele as the ancestral allele, as Relate software [18] has the capability to adjust ancestral alleles during analysis.

### Constructing ARG and estimating relative coalescence rates through time

We constructed the ARG using Relate (v1.1.9) [18], applied to 689 phased whole-genome sequences. The following parameters were used: mutation rate: 1.26 × 10^−8^ per base per generation; recombination rate: 1.00 × 10^−8^; effective population size (*Ne*): 10,000; seed: 1. Subsequently, we used Relate’s EstimatePopulationSize.sh script to estimate coalescence rates and re-estimate branch lengths. The parameters for this step were: mutation rate: 1.26 × 10^−8^; generation time: 6 years; number of iterations: 10; tree dropping threshold: 0.5; painting: 0.025 1; time bins: defined as 10*^x^* years ago, where *x* ranges from 2 to 6.6 with an increment of 0.1.

To infer relative cross-coalescence rates between the five beef cattle breeds, we extracted subtrees such that all tips belonged to these five breeds and re-estimated coalescence rates. We then extracted coalescence rates and cross-coalescence rates from the .coal files to calculate relative cross-coalescence rates.

### *F*st and CLUES analyses

We grouped Angus and Hereford as one population and Charolais, Simmental, and Limousin as another population. Using VCFtools (v0.1.16) [50], we performed *F*st analysis on each SNV in the ARG for these five beef cattle breeds. The top 0.05% of SNVs with the highest genome-wide *F*st values were considered highly differentiated and selected for subsequent CLUES analysis. To reduce false positives caused by potentially inaccurate ARG inference in some local regions, we applied the following filters to remove SNVs: (1) SNVs with the number of mutations mapping to their tree in the bottom 5th percentile; and (2) SNVs where the fraction of tree branches having at least one SNP is in the bottom 5th percentile.

We extracted ARG of each breed separately and re-estimated coalescence rates and branch lengths to serve as input for the CLUES selection test [19]. For each highly differentiated SNV, we first used Relate’s SampleBranchLengths.sh to extract the corresponding local tree and resampled branch lengths 200 times. CLUES calculates an LR for each input SNV, reflecting the degree to which the derived allele of this SNV deviates from the neutral mutation model. We repeated the calculation three times for each highly differentiated SNV using different seeds (seed = 1, 10, 100). The median of the log LR values was then assigned to each SNV.

### CLUES selection test adjusted for genetic drift in population history

To reduce false positives caused by genetic drift and determine the threshold for rejecting neutral mutations in CLUES analysis, we simulated a series of neutral mutations based on the effective population size changes over time, as estimated by Relate for the five beef cattle breeds. We calculated the changes in effective population size over time based on the coalescence rates estimated by Relate for the five beef cattle breeds. As all five beef cattle breeds are of European origin, their effective population size trends were generally consistent. We used the average effective population size of the five breeds for subsequent data simulation. The msprime (v1.2.0) [51] was applied to simulation with parameters: sequence length: 158,534,110 bp (length of chromosome 1 in ARS-UCD1.2); number of individuals: 100 (200 haplotypes), matching each beef cattle breed; recombination rate: 1 × 10^−8^; mutation rate: 1.26 × 10^−8^; random seed: 1; model: Classical coalescent with recombination model (Hudson’s algorithm)

We converted all simulated biallelic SNVs into Relate input format and inferred the ARG using the same parameters as for the cattle ARG construction. The effective population size changes inferred from the simulated data showed the same trend as the real data within ∼ 300,000 years (the divergence time between taurine and zebu cattle). After removing low-quality sites, we obtained 1,006,489 SNVs. We selected one SNV every 100 positions, resulting in 10,064 SNVs for the CLUES selection test, following the same procedure as for the real data. The resulting log LR distribution from simulated data, with 95%, 99%, and 99.9% percentiles, is shown in Figure S2.

### Genome coordinate remapping

To ensure consistency with our analysis based on the ARS-UCD1.2 reference genome, we performed coordinate remapping for two sets of previously published data. First, we addressed the GWAS result for daily weight gain in beef cattle conducted by Zhang and colleagues [12]. In their study, the physical positions of variants were based on the UMD3.1 reference genome. To align these coordinates with our current framework, we utilized NCBI’s Remap Tool to reposition the UMD3.1-based physical coordinates onto the ARS-UCD1.2 reference genome.

Secondly, we focused on the cattle stature QTLs reported by Bouwman and colleagues [2]. Similar to the previous dataset, the genomic coordinates for these QTLs were originally based on the UMD3.1 reference genome. For this remapping, we employed the UCSC LiftOver tool [52] to translate the UMD3.1-based physical coordinates to their corresponding positions on the ARS-UCD1.2 reference genome.

### Mapping and variant calling for ancient cattle genomic data

Our ancient cattle genomic data were sourced from the study by Verdugo and colleagues [53]. We followed their methodology for processing the ancient cattle genome sequencing data, with some modifications. The specific steps were as follows:

We first used Cutadapt [54] to remove adapters from reads and filter out low-quality reads, using the following parameters: -a AGATCGGAAGAGCACACGTCTGAACTCCAGTCAC -O 1 -m 30. We then aligned the quality-controlled reads to the ARS-UCD1.2 reference genome using the aln module of bwa (v0.7.17) [55], and used the samse module with the option “-r” for defining read groups and producing unfiltered SAM files. We used SAMtools (v1.14) [56] for quality control and sorting of reads in the SAM files, generating BAM files (with parameters: -Sb -F 4 -q 25), followed by duplicate removal. Picard tools (v2.16.0) was used to merge BAM files. INDEL realignment was performed using GATK (v3.8-0-ge9d806836) [57]. For samples Th7 and Ch22, we removed duplicates using Picard tools (v2.16.0) before performing INDEL realignment.

For all autosomes, we used BCFtools’s mpileup and call modules (bcftools call -m) to call SNVs on the filtered ancient cattle BAM files, based on the positions of biallelic SNVs from Run 9 of the 1000 Bull Genomes Project. The variant rs384548488 (Chr6:g.37,401,771_37,401,772del) is an INDEL. To obtain the genotypes of rs384548488 and nearby SNVs in ancient cattle, we performed genotyping on all sites in the genomic region Chr6:37,151,770–37,651,770 using BCFtools’s mpileup and call modules (bcftools call -m).

### Mapping and variant calling for modern genomic data

For the downloaded FASTQ files, we used fastp (v0.23.4) [58] with default parameters to remove adapters and low-quality reads. The processed reads were then analyzed following the GATK Best practices for data pre-processing for variant discovery and somatic short variant discovery (SNVs + INDELs). In brief, we first used the MEM module of BWA to map reads to the reference genome. Subsequently, we used SAMtools to sort the BAM files and Picard tools to remove duplicates. GATK HaplotypeCaller, CombineGVCFs, and GenotypeGVCFs were employed for joint genotyping of multiple samples.

### Prediction of variant effects

To annotate variants in cattle, we utilized Variant Effect Predictor (VEP) [59] from Ensembl (release 112) and cache (v110) to predict variant effects. We assigned 5 kb as the distance up and/or downstream between a variant and a transcript for which VEP will assign the upstream_gene_variant or downstream_gene_variant consequences. Additionally, we employed ANNOVAR [60] to annotate variants located in intergenic regions. We defined intergenic regions as being the same if they share the nearest upstream and downstream genes.

### Scanning pLoPD variants

To identify potential variants causing the loss of the PIP domain in PALI2, we first obtained the amino acid sequences of the PIP domain for cattle, rabbit, and chicken PALI2 proteins from the study by Conway and colleagues [22]. For sheep, goat, horse, dog, and pig, where known PALI2 PIP domain sequences were unavailable, we used the cattle PALI2 PIP domain sequence as a proxy. We then employed NCBI’s online BLAST tool to align these PIP domain amino acid sequences to the reference genomes of sheep and pig, thereby determining the genomic coordinates of the PALI2 PIP domain in these species. The reference genomes used for cattle, sheep, goat, pig, horse, dog, rabbit, and chicken were ARS-UCD1.2, ARS-UI_Ramb_v2.0, ARS1, Sscrofa11.1, Equcab3.0, ROS_Cfam_1.0, OryCun2.0, and GRCg7w, respectively. For gene annotations, we utilized Ensembl (release 104) for ARS-UCD1.2 and ROS_Cfam_1.0 and Ensembl (release 112) for ARS-UI_Ramb_v2.0, ARS1, Sscrofa11.1, and OryCun2.0. As Ensembl annotations were not available for the GRCg7w genome, we used NCBI gene annotations for chicken.

Rice et al. published a pangenome reference (GRCg7b) constructed from 30 chickens [28]. However, neither Ensembl nor NCBI gene annotations for GRCg7b fully annotated the mRNAs and exons of *LCORL* containing the PIP domain. Consequently, we couldn’t directly assess the impact of variants on the PALI2 PIP domain based on GRCg7b annotations. Therefore, we aligned the genomic coordinates of variants based on GRCg7b to GRCg7w using BLAST, allowing us to evaluate the potential effects of these variants on the PALI2 PIP domain based on NCBI gene annotations for the GRCg7w genome.

For sheep, pig, and rabbit, we obtained the ancestral sequences near the *LCORL* frameshift intron from the Ensembl 43 eutherian mammals EPO multiple alignment data [61].

### Haplotype and genotype pattern analyses

For cattle, pig, and sheep, to analyze the haplotype patterns composed of both INDELs and SNVs, we employed BCFtools (v1.16) to filter variants (see File S1 Section 2 for detailed filtering parameters and processes). After filtering, we used Beagle (v5.4; with parameters: window = 10, overlap = 2) to phase SNVs and INDELs for cattle, pig, and sheep variants. For all species studied, we extracted mutations within the *LCORL* locus. To visualize the haplotype or genotype patterns, we developed custom scripts to generate heatmaps. The bifurcation diagram for haplotypes was plotted using R package rehh [62].

### Motif prediction

To predict transcription factor binding sites, we employed the Motif Alignment and Search Tool (MAST) from the MEME Suite (v5.5.5) [63]. Our approach began with the extraction of the sequence of the active regulatory element (Chr20:4,967,400–4,969,800) containing rs110540352 from the reference genome using SAMtools (v1.16). We then utilized the Position Frequency Matrix (PFM) data for vertebrates from the JASPAR CORE database 2024 [64] as our motif database. Using MAST with its default parameters, we scanned the extracted sequence for potential transcription factor binding sites. This analysis was performed twice: once for the sequence containing the ancestral allele at rs110540352, and once for the sequence containing the derived allele. By comparing the MAST results for both the reference and alternate sequences, we were able to predict how the rs110540352 variant might affect transcription factor binding within this active regulatory element.

### Luciferase assays

The ancestral *STC2* regulatory element fragment (5′-AAGTGGCGCCTCAA-3′), the mutational *STC2* regulatory element fragment (5′-AAGTGGCGCCTGAA-3′), and the E2F4 coding sequence (ENSBTAT00000015999.7) were synthesized by Sangon Biotech (Shanghai, China). The E2F4 coding sequence was inserted into pCDNA3.1_3XFlag (Catalog No. 182494, Addgene, Watertown, MA), named pCDNA3.1_3XFlag-E2F4. The ancestral *STC2* regulatory element fragment and the mutational *STC2* regulatory element fragment were inserted into the luciferase reporter vector pGL4.10, named pGL4.10-ANC and pGL4.10-MUT, respectively.

HEK293T cells [Catalog No. CRL-1573, American Type Culture Collection (ATCC), Manassas, VA] were cultured in Dulbecco’s Modified Eagle Medium (DMEM; Catalog No. 11965092, Gibco, Waltham, MA) supplemented with 10% fetal bovine serum (FBS; Catalog No. F101, Gibco) at 38.5°C in a 5% CO_2_ environment. Approximately 1 μg of plasmid DNA (0.45 μg each for pGL4.10-ANC or pGL4.10-MUT, 0.45 μg for pCDNA3.1_3XFlag-E2F4, and 0.1 μg for pRL-TK) was cotransfected using PEI Transfection Reagent (Catalog No. HY-K2014, MCE, Monmouth Junction, NJ) according to the manufacturer’s instructions. The pRL-TK plasmid served as an internal control for normalizing transfection efficiency. Cells were harvested and lysed 48 h post transfection and prepared for luciferase activity analysis using the Double-Luciferase Reporter Assay Kit (Catalog No. FR201, TransGen Biotech, Beijing, China) following the manufacturer’s instructions. Relative luciferase activities were expressed as the ratio of firefly luciferase to Renilla luciferase values.

### Quantification of allele expression

In Hereford cattle, rs110540352*G and rs42661323*G are in high LD. The rs110540352(C>G) is located in an intergenic region, while rs42661323(C>G) is situated in an exon of the *STC2* gene. To investigate the effect of rs110540352(C>G) on *STC2* expression in the hypothalamus and cerebral cortex, we counted the reads carrying rs42661323*C and rs42661323*G in individuals heterozygous for rs42661323. By analyzing the potential differences between reads carrying rs42661323*C and rs42661323*G, we inferred the impact of rs110540352(C>G) on *STC2* expression.

We selected RNA-seq data from the cerebral cortex of Hereford cattle from the CattleGTEx dataset. To process the data, we used fastp (v0.23.4) with parameters of “cut_window_size 4” and “cut_mean_quality 15” to remove adapters and low-quality reads. Subsequently, we employed Hisat2 [65] with default parameters to map the RNA-seq reads to the ARS-UCD1.2 reference genome and used SAMtools (v1.16) to sort the reads. We further utilized Picard toolkit (v3.0.0) to remove duplicates.

For visualization and quantification, we used IGV (v11.0.1) [66] to inspect the BAM files and count the reads carrying rs42661323*C and rs42661323*G. Due to the relatively low number of reads obtained from each sample, we combined the counts of reads carrying rs42661323*C and rs42661323*G from all samples for our analysis.

### Functional validation of LCORL lacking the PIP domain in mice

We performed sequence alignment of the exons encoding the PIP domain of LCORL in cattle and mice using the MUSCLE algorithm in MEGA (v11) [67]. This analysis identified the homologous position of the cattle rs384548488 variant in mice as Chr5:45,882,519–45,882,520 (Figure S6A). We targeted the region Chr5:45,882,459–45,882,558 for CRISPR/Cas9-mediated deletion to induce a frameshift mutation, which was carried out by Cyagen Biosciences Inc. (Suzhou, China). Briefly, two gRNAs targeting the mouse *Lcorl* gene (gRNA-A1 matching the reverse strand: 5′-AGGGTTAAAAGATTCATTTTGGG-3′, and gRNA-A2 matching the forward strand: 5′-TTGTCACTGTTGTTTATGGAAGG-3′) were co-injected with Cas9 mRNA into fertilized C57BL/6J mouse oocytes to generate offspring carrying the targeted gene deletion. F0 founder animals were identified through PCR [forward primer (F1): 5′-CAAGAAGACCCTAAGGAAAAGTCA-3′, reverse primer (R1): 5′-TCTGAGGTATCATAGACTTGCTCT-3′] followed by sequencing. These animals were subsequently mated with wild-type mice to test germline transmission and produce F1 offspring.

We commissioned Cyagen Biosciences Inc. to perform *in vitro* fertilization (IVF) using heterozygous male and female mice to rapidly generate an F2 cohort (*n* = 112; 56 males and 55 females). All mice were housed under *ad libitum* feeding conditions, with no more than 5 mice per cage. We commissioned Cyagen Biosciences Inc. to measure the body weight of 10 individuals per genotype for both male and female mice, starting from weaning (3 weeks of age). All mice undergoing weight measurements were housed in groups of 5 per cage under identical environmental conditions with free access to food. Data are represented by mean ± standard error (SE). Differences between means were assessed by two-tailed Student’s *t*-test.

To evaluate the potential off-target effects, we utilized CCTop [68] to computationally predict potential off-target sites for each gRNA. We performed whole-genome resequencing on three F1 *Pali2*^+/−^ and *Stc2*^A60P/A60P^ mice. The results showed no off-target effects at the top 5 potential off-target sites for the 3 gRNAs (Figure S6).

### Functional validation of STC2 A60P in mice

We commissioned Cyagen Biosciences Inc. to generate the STC2 A60P mice via CRISPR/Cas9-mediated gene editing. Briefly, a gRNA targeting the mouse *Stc2* gene (matching the forward strand of *Stc2*: 5′-CAGCACTGTTTGGTCAATGCCGG-3′), a donor oligonucleotide containing the p.A60P (GCC to CCC) mutation, and Cas9 were co-injected into fertilized C57BL/6J mouse oocytes to produce offspring carrying the targeted gene knock-in. F0 founder animals were identified through PCR [forward primer (F1): 5′-CGATAGAAGGAAGAAAAGAAAACGC-3′, reverse primer (R1): 5′-CAAACACCAGACTCTCTCAAGCAA-3′] followed by sequencing. These animals were subsequently mated with wild-type mice to test germline transmission and produce F1 offspring. We commissioned Cyagen Biosciences Inc. to perform IVF using heterozygous male and wild-type female mice to generate 30 heterozygous males and 30 heterozygous females rapidly. We then bred these 30 pairs of heterozygous mice to produce the F2 generation (*n* = 126; 70 males and 56 females). Body weight measurements were conducted on 125 mice. All mice were housed in groups of 5 per cage under identical environmental conditions with free access to food. Data are represented by mean ± SE. Differences between means were assessed by two-tailed Student’s *t*-test.

### Total protein extraction and Western blot

The embryos and thymus and muscle tissues from control and mutant groups were washed with ice-cold phosphate-buffered saline (PBS). Subsequently, they were homogenized using 3-mm stainless steel beads in RIPA Lysis Buffer (Catalog No. R0020, Solarbio, Beijing, China) supplemented with 1% protease inhibitor cocktail (Catalog No. 05056489001, Roche, Basel, Switzerland) and 1 mM phenylmethylsulfonyl fluoride (PMSF; Catalog No. ST506, Beyotime, Shanghai, China) to extract total proteins.

After measuring protein concentration with BCA kit (Catalog No. ZJ102, Epizyme, Shanghai, China), protein samples were denatured by boiling in 1× Protein Loading Buffer (Catalog No. LT101S, Epizyme) and separated on 10% sodium dodecyl sulfate-polyacrylamide gel electrophoresis (SDS-PAGE) gels (Catalog No. PG212, Epizyme). The separated proteins were then transferred to polyvinylidene fluoride (PVDF) membranes by wet transfer. The membranes were blocked with 5% nonfat milk for 2 h at room temperature and subsequently incubated with primary antibodies at 4°C overnight. After washing, the membranes were incubated with HRP-coupled secondary antibodies for 2 h at room temperature. Finally, protein bands were visualized with a chemiluminescence detection system. The antibodies used are as follows: anti-H3K27me3 (1:1000; Catalog No. 9733s, Cell Signaling Technology, Danvers, MA), anti-β-actin (1:1000; Catalog No. 8457, Cell Signaling Technology), and anti-β-tubulin (1:1000; Catalog No. 2128S, Cell Signaling Technology).

## Ethical statement

Animal experiments were performed in accordance with the regulations and guidelines established by the Animal Care Committee of Northwest A&F University, China (Approval No. XN-20230529).

## Supplementary Material

qzaf025_Supplementary_Data

## Data Availability

Relate-estimated coalescence rates and allele ages for the Charolais, Simmental, Limousin, Hereford, Angus cattle have been deposited at Zenodo (https://zenodo.org/records/14259711). Our predictions of cattle ancestral alleles for SNVs have been deposited at Zenodo (https://zenodo.org/records/14261687). The RNA-seq and DNA-seq data of mice generated in this study have been deposited in the Genome Sequence Archive [69] at the National Genomics Data Center (NGDC), China National Center for Bioinformation (CNCB) (BioProject: PRJCA036933), and are publicly accessible at https://ngdc.cncb.ac.cn/gsa.

## CRediT author statement

**Fengting Bai:** Conceptualization, Data curation, Formal analysis, Methodology, Software, Visualization, Writing – original draft. **Yudong Cai:** Conceptualization, Formal analysis, Methodology, Software, Writing – review & editing. **Min Qiu:** Formal analysis, Visualization. **Chen Liang:** Investigation. **Linqian Pan:** Investigation. **Yayi Liu:** Investigation. **Yanshuai Feng:** Investigation. **Xuesha Cao:** Investigation. **Qimeng Yang:** Visualization. **Gang Ren:** Validation. **Shaohua Jiao:** Data curation. **Siqi Gao:** Investigation. **Meixuan Lu:** Investigation. **Xihong Wang:** Funding acquisition, Writing – review & editing. **Rasmus Heller:** Writing – review & editing. **Johannes A. Lenstra:** Writing – review & editing. **Yu Jiang:** Conceptualization, Funding acquisition, Methodology, Project administration, Resources, Supervision, Validation, Writing – review & editing. All authors have read and approved the final manuscript.

## ORCID

0009-0007-6907-6758 (Fengting Bai)

0000-0001-5902-0901 (Yudong Cai)

0009-0006-8732-6981 (Min Qiu)

0009-0008-1956-0207 (Chen Liang)

0009-0005-6473-2002 (Linqian Pan)

0009-0004-2937-8872 (Yayi Liu)

0009-0009-3241-7410 (Yanshuai Feng)

0009-0005-5153-1981 (Xuesha Cao)

0000-0002-9830-2466 (Qimeng Yang)

0000-0001-9690-5995 (Gang Ren)

0009-0005-4659-7636 (Shaohua Jiao)

0009-0003-3520-795X (Siqi Gao)

0009-0006-6159-9290 (Meixuan Lu)

0000-0002-8277-969X (Xihong Wang)

0000-0001-6583-6923 (Rasmus Heller)

0000-0003-1745-7739 (Johannes A. Lenstra)

0000-0003-4821-3585 (Yu Jiang)
