# Supplementary material for: LCORL and STC2 Variants Increase Body Size and Growth Rate in Cattle and Other Animals
Source: Genomics Proteomics Bioinformatics. 2025 Mar 17;23(3):qzaf025. doi: 10.1093/gpbjnl/qzaf025 (PMC12448305; doi:10.1093/gpbjnl/qzaf025)
Supplement: qzaf025_Supplementary_Data [file qzaf025_supplementary_data.zip › File_S1.docx]

**File S1**

**Section 1 Individual selection for ARG construction**

To retain a larger number of unrelated individuals within each breed, we employed the following strategies:

For Angus, Hereford, and Charolais:

We iteratively removed individuals with the most third-degree or closer relationships (kinship coefficient > 0.0442) within each breed until all remaining individuals were unrelated. We then retained the 100 individuals with the highest sequencing depth.

For Simmental:

We iteratively removed individuals with the most third-degree or closer relationships (kinship coefficient > 0.0442) within each breed until all remaining individuals were unrelated. This process yielded 81 Simmental individuals. We applied the same method to select 19 German Simmental (Fleckvieh) individuals, which were then combined with the Simmental group to reach a total of 100.

For Limousin:

Due to the limited sample size of 101, we only removed the individual with the lowest sequencing depth, retaining 100 individuals.

**Section 2 Variant filtering for haplotype and genotype pattern analysis**

For cattle, we retained SNPs and INDELs with a missing rate of less than 20%. Variants from the GATK VQSR (Variant Quality Score Recalibration) 99.90 to 100.00 Tranche for both SNPs and INDELs were excluded from our analysis.

For pig, sheep, we applied the GATK-recommended hard filtering thresholds to filter INDELs and SNVs. Specifically, for SNVs, we used the following criteria: INFO/QD >= 2.0 & QUAL >= 30.0 & INFO/SOR <= 3.0 & INFO/FS <= 60.0 & INFO/MQ >= 40.0 & MQRankSum >= -12.5 & INFO/ReadPosRankSum >= -8.0. For INDELs, we applied these thresholds: INFO/QD >= 2.0 & QUAL >= 30.0 & INFO/FS <= 200.0 & INFO/ReadPosRankSum >= -20.0.
