# Supplementary figures and images for: LCORL and STC2 Variants Increase Body Size and Growth Rate in Cattle and Other Animals
Source: Genomics Proteomics Bioinformatics. 2025 Mar 17;23(3):qzaf025. doi: 10.1093/gpbjnl/qzaf025 (PMC12448305; doi:10.1093/gpbjnl/qzaf025)

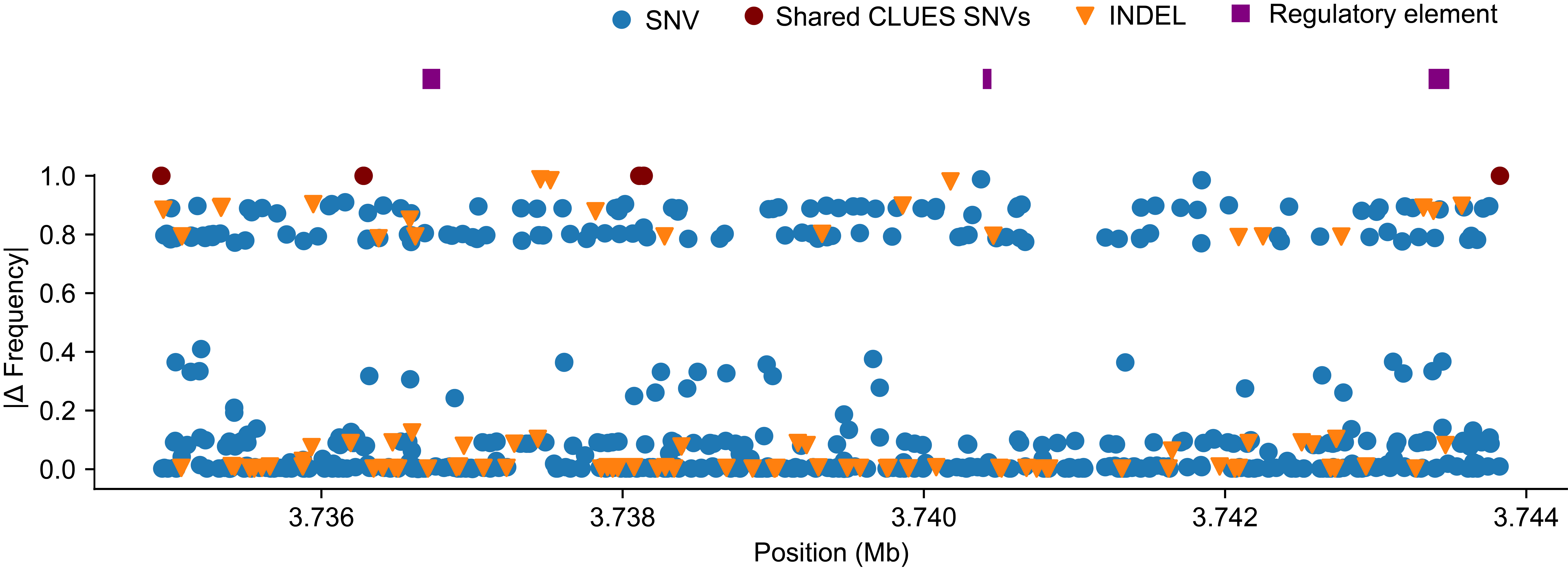

Supplement: qzaf025_Supplementary_Data [file qzaf025_supplementary_data.zip › Figure_S4.jpg]

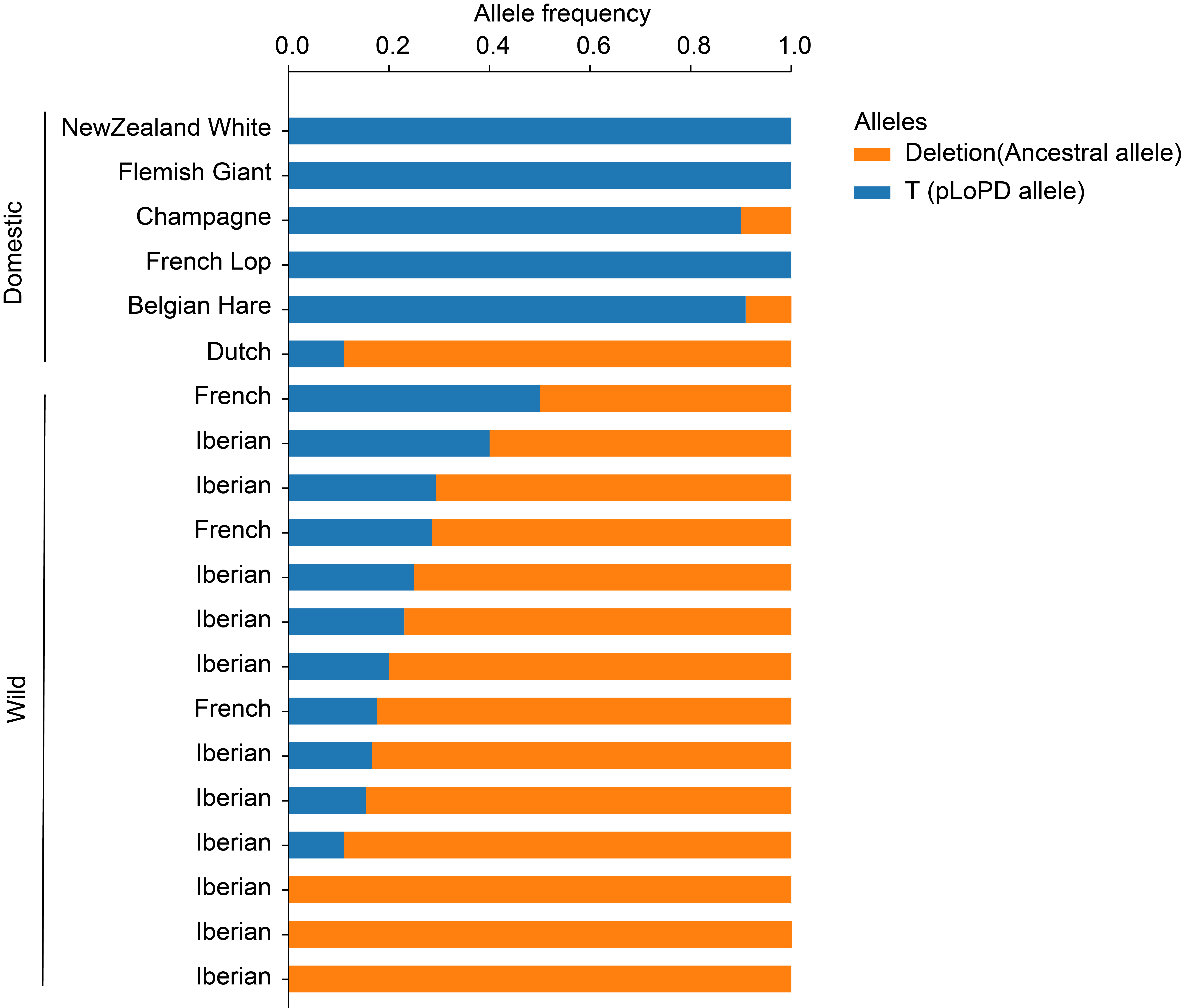

Supplement: qzaf025_Supplementary_Data [file qzaf025_supplementary_data.zip › Figure_S12.jpg]

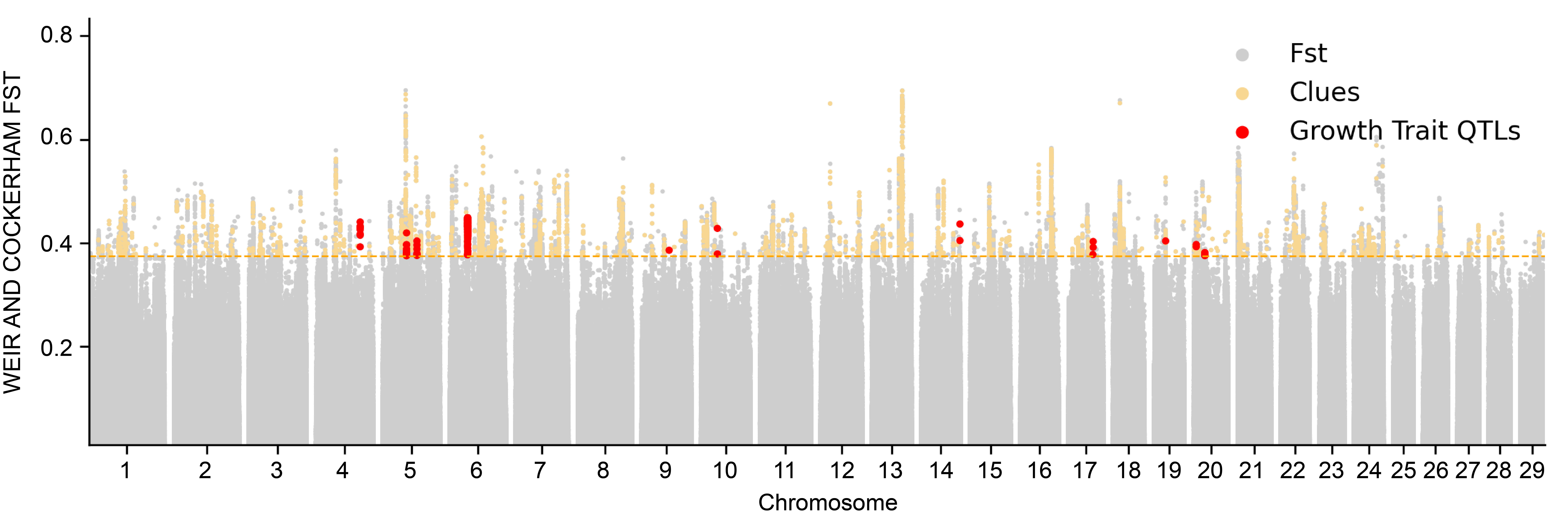

Supplement: qzaf025_Supplementary_Data [file qzaf025_supplementary_data.zip › Figure_S1.jpg]

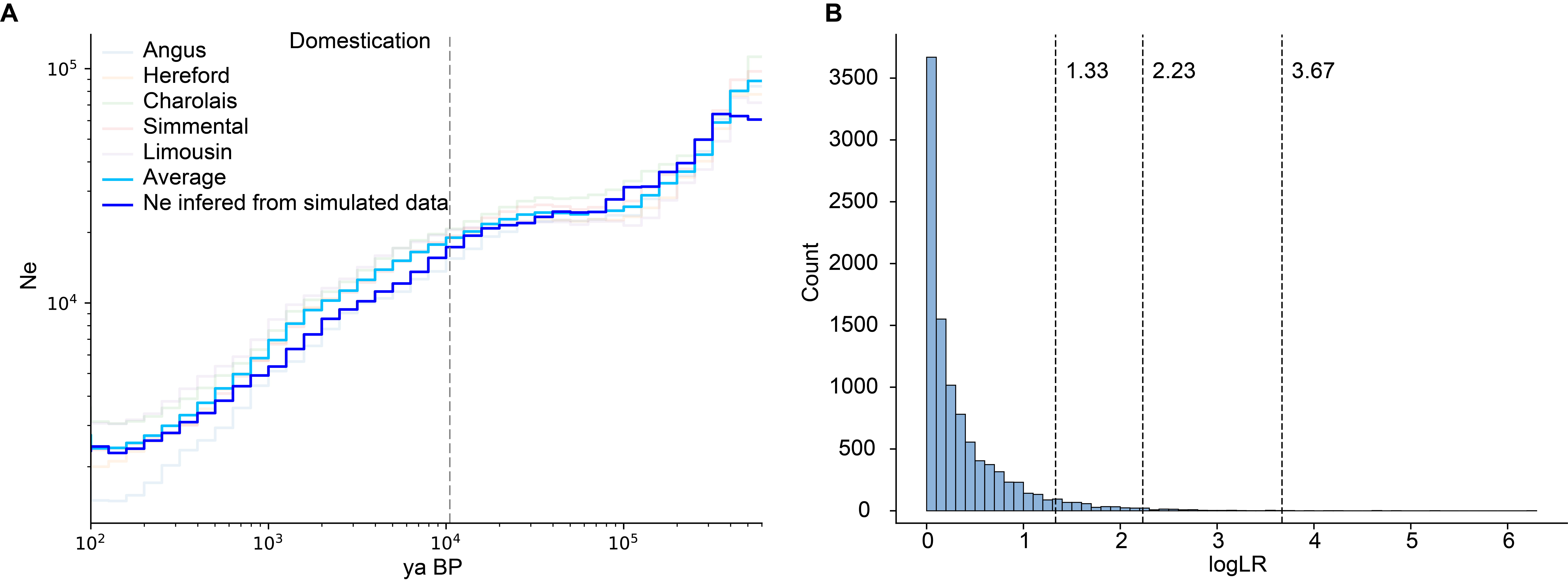

Supplement: qzaf025_Supplementary_Data [file qzaf025_supplementary_data.zip › Figure_S2.jpg]

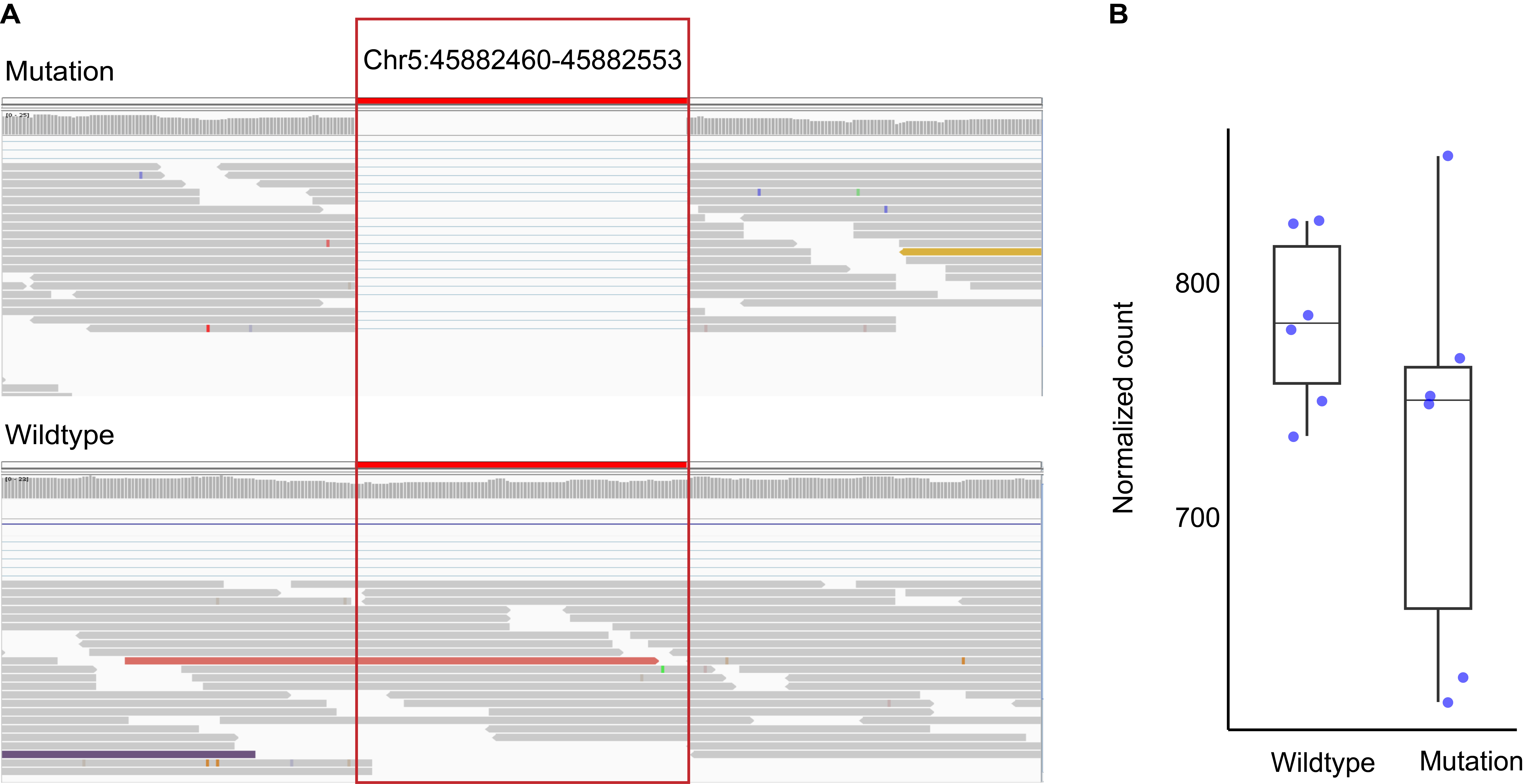

Supplement: qzaf025_Supplementary_Data [file qzaf025_supplementary_data.zip › Figure_S8.jpg]

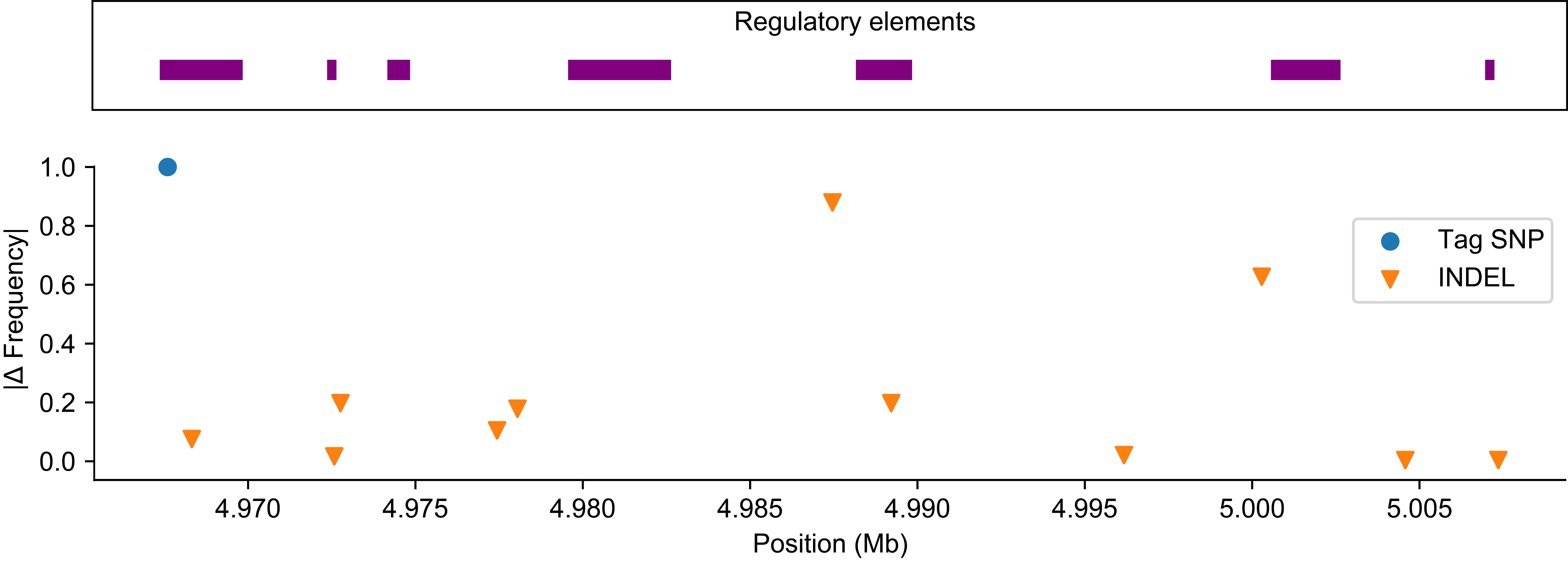

Supplement: qzaf025_Supplementary_Data [file qzaf025_supplementary_data.zip › Figure_S17.jpg]

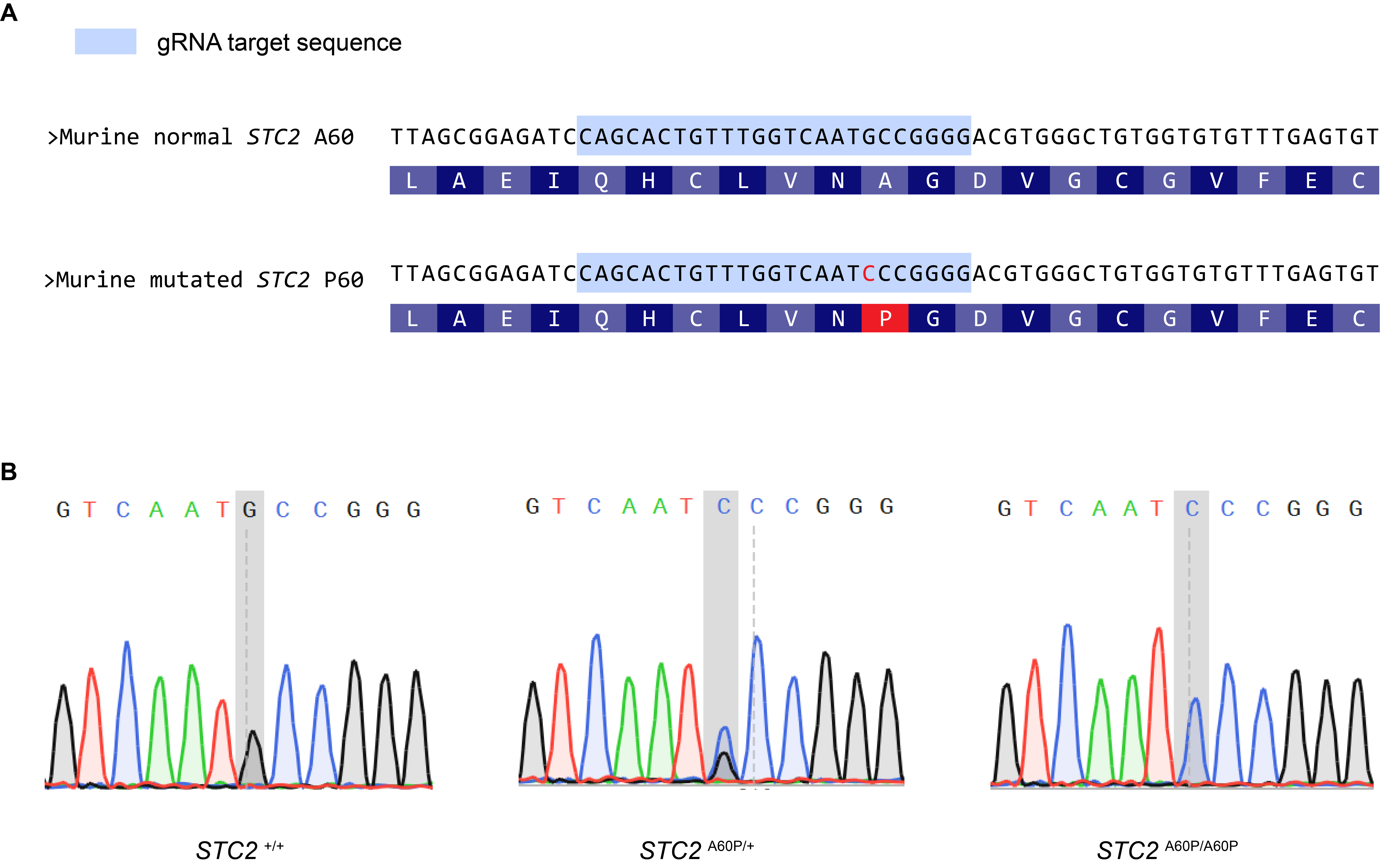

Supplement: qzaf025_Supplementary_Data [file qzaf025_supplementary_data.zip › Figure_S19.jpg]
